# Gene expression during normal and FSHD myogenesis

**DOI:** 10.1186/1755-8794-4-67

**Published:** 2011-09-27

**Authors:** Koji Tsumagari, Shao-Chi Chang, Michelle Lacey, Carl Baribault, Sridar V Chittur, Janet Sowden, Rabi Tawil, Gregory E Crawford, Melanie Ehrlich

**Affiliations:** 1 Human Genetics Program, Tulane Medical School, New Orleans, LA, USA; 2 Department of Mathematics, Tulane University, New Orleans, LA, USA; 3 Tulane Cancer Center, New Orleans, LA, USA; 4 Biomedical Sciences, University at Albany-SUNY, Albany, NY, USA; 5 Department of Neurology, University of Rochester School of Medicine and Dentistry, Rochester, NY, USA; 6 Institute for Genome Sciences & Policy, Duke University, Durham, NC, USA

## Abstract

**Background:**

Facioscapulohumeral muscular dystrophy (FSHD) is a dominant disease linked to contraction of an array of tandem 3.3-kb repeats (D4Z4) at 4q35. Within each repeat unit is a gene, *DUX4*, that can encode a protein containing two homeodomains. A *DUX4 *transcript derived from the last repeat unit in a contracted array is associated with pathogenesis but it is unclear how.

**Methods:**

Using exon-based microarrays, the expression profiles of myogenic precursor cells were determined. Both undifferentiated myoblasts and myoblasts differentiated to myotubes derived from FSHD patients and controls were studied after immunocytochemical verification of the quality of the cultures. To further our understanding of FSHD and normal myogenesis, the expression profiles obtained were compared to those of 19 non-muscle cell types analyzed by identical methods.

**Results:**

Many of the ~17,000 examined genes were differentially expressed (> 2-fold, *p *< 0.01) in control myoblasts or myotubes vs. non-muscle cells (2185 and 3006, respectively) or in FSHD vs. control myoblasts or myotubes (295 and 797, respectively). Surprisingly, despite the morphologically normal differentiation of FSHD myoblasts to myotubes, most of the disease-related dysregulation was seen as dampening of normal myogenesis-specific expression changes, including in genes for muscle structure, mitochondrial function, stress responses, and signal transduction. Other classes of genes, including those encoding extracellular matrix or pro-inflammatory proteins, were upregulated in FSHD myogenic cells independent of an inverse myogenesis association. Importantly, the disease-linked *DUX4 *RNA isoform was detected by RT-PCR in FSHD myoblast and myotube preparations only at extremely low levels. Unique insights into myogenesis-specific gene expression were also obtained. For example, all four Argonaute genes involved in RNA-silencing were significantly upregulated during normal (but not FSHD) myogenesis relative to non-muscle cell types.

**Conclusions:**

*DUX4*'s pathogenic effect in FSHD may occur transiently at or before the stage of myoblast formation to establish a cascade of gene dysregulation. This contrasts with the current emphasis on toxic effects of experimentally upregulated *DUX4 *expression at the myoblast or myotube stages. Our model could explain why *DUX4*'s inappropriate expression was barely detectable in myoblasts and myotubes but nonetheless linked to FSHD.

## Background

Differentiation of myoblasts to myotubes is one of the best cell culture models for vertebrate differentiation. However, there has been only limited expression profiling of well characterized myoblast cell strains and of myoblasts differentiated in vitro to myotubes [1-3]. In this study, we profiled expression of control myoblasts and myotubes as well as analogous cells from patients with facioscapulohumeral muscular dystrophy (FSHD). Importantly, we were able to compare control and FSHD myoblasts and myotubes with 19 different non-muscle cell types subjected to identical expression profiling. The data are directly comparable because the same experimental and computational techniques were used for all the cell types. This allowed us to identify myogenesis-specific as well as disease-associated differences in expression. We are particularly interested in regenerative myogenesis [4], as opposed to embryonic myogenesis [5], because of its role in limiting atrophy due to muscle damage, aging, and disease.

FSHD is a dominant disease whose pathogenesis is still perplexing despite new insights into its genetic linkage [6-8]. It is progressively debilitating and painful and mainly affects skeletal muscle. FSHD is linked to contraction at 4q35 of a tandem array of 3.3-kb repeats, D4Z4, from about 11-100 to 1-10 copies [9]. It is usually diagnosed in the second decade, and the patient's lifespan is generally not affected. Initially, the pathology is limited to a small set of skeletal muscles, often asymmetrically. There is apparently no involvement of smooth muscle. No efficacious treatment is available.

Although other expression profiling studies of FSHD vs. normal- or disease-control muscle biopsies have been done [6,10-13], no clear consensus has emerged as to the genes that lead to the muscle pathology. Usually, only modest up- or downregulation of gene expression was observed. FSHD is likely to involve defects in muscle cell precursors [10]; therefore, studies of FSHD myoblasts and myotubes should also elucidate normal myogenesis. In analyses of muscle tissue, myogenesis-specific, disease-related changes in expression are obscured by the very low percentages of (activated) satellite cells. Upon expression profiling of FSHD and control myoblasts (but not myotubes) in 2003, Winokur et al. found ~20 genes were FSHD-dysregulated; among them were genes involved in the response to oxidative stress [14]. Accordingly, they demonstrated and Barro et al. confirmed [14,15] that FSHD myoblasts are significantly more sensitive to the lethal effects of drug-induced oxidative stress than normal-control and disease-control myoblasts. Nonetheless, Barro et al. demonstrated that this hypersensitivity did not affect growth rates or the ability of myoblasts to differentiate to myotubes. A recent expression profiling study of FSHD and control myoblasts and myotubes by Cheli et al. [16] provided no characterization of the purity of the myoblast or myotube samples and paradoxically reported no muscle-related terms among 177 functional terms for genes with differential expression in normal-control myoblast vs. normal-control myotube preparations, which is very different from what we have found, as described below.

A number of 4q35 genes have been considered as candidates for the initially dysregulated gene during FSHD pathogenesis, namely, *FRG1, DUX4, DUX4C, ANT1 (SLC25A4), FRG2, TUBB4Q*, and *FAT1 *[6,17-24]. Recently implicated in FSHD pathogenesis from genetic mapping is *DUX4*, a 1.6-kb gene that resides within each 3.3-kb repeat unit of D4Z4. *DUX4 *encodes a protein containing two homeodomains [25,26]. The protein is strongly pro-apoptotic when highly overexpressed in experimental models [21,27-29]. *DUX4 *transcripts are normally difficult to detect probably because of heterochromatinization of normal long D4Z4 arrays inhibiting their transcription [30,31] and the lack of a polyadenylation signal within *DUX4 *[32], which generally leads to *DUX4 *mRNA being unstable. However, in patients, a polyadenylation signal is provided for the most distal *DUX4 *gene in the D4Z4 array by a common SNP located immediately distal to D4Z4 and specific to 4q35, but not to the non-pathogenic 10q26 D4Z4 array [6,7]. Therefore, expression of the FSHD-linked *DUX4 *RNA isoform, *DUX4-fl *(full length), generally requires both array contraction at 4q35 and this SNP. Exceptions to the requirement for array contraction for generation of detectable *DUX4-fl *transcript were seen in normal testis and in myoblasts and myotubes from patients with a variant of FSHD called FSHD2, which is associated with inappropriate expression of *DUX4-fl *RNA from 4q35 despite a lack of contraction of the D4Z4 array [8]. Both of these exceptions may involve D4Z4 chromatin loosening in normal-length arrays due to partial hypomethylation of D4Z4 DNA [31,33].

*DUX4-fl *RNA was found in all examined FSHD myotube preparations, several FSHD myoblast cell strains, and two FSHD fibroblast cell strains, but not in the analogous control cells [7,8]. However, there are still fundamental questions about the disease mechanisms. Firstly, in view of the convincing genetic data linking FSHD to the *DUX4-fl *RNA isoform [6-8], why was *DUX4-fl *RNA expression in FSHD myoblasts and myotubes extraordinarily infrequent, e.g., ~ 1 in 1000 myoblast nuclei positive [8]? Why did two of five FSHD myoblast preparations lack detectable *DUX4-fl *RNA by nested RT-PCR [8] despite the finding that FSHD myoblasts have a phenotype of hypersensitivity to oxidative stress [14,15]? Why is FSHD essentially only a muscle-specific disease even though FSHD fibroblasts and FSHD myotubes display similar (very low) levels of *DUX4-fl *RNA isoform [8]? And lastly, why do C2C12 myoblasts with induced expression of human *D4Z4-fl *RNA [27,28] undergo apoptosis or display diminished differentiation to myotubes, unlike FSHD myoblasts, which grow and differentiate normally? Although apoptosis has been noted in FSHD muscle samples, it was mostly in late-stage muscle biopsies [34].

In the current study, using exon-based expression microarrays, we found differences in expression of diverse categories of genes in control myoblasts and myotubes relative to various non-muscle cell types. In addition, FSHD muscle progenitors had a unique pattern of gene dysregulation compared with analogous control cells. A distinctive expression profile was observed in FSHD myoblasts as well as in FSHD myotubes even though *DUX4-fl *RNA was present at extremely low levels or was undetectable in these cells.

## Methods

### Cell culture

Myoblast cell strains from FSHD patients and controls and the other cell types are described in Additional File 1, Additional File 2, and Additional File 3. FSHD myoblast cell strains from moderately affected muscle were no more difficult to generate than control myoblast cell strains. Duly signed patient consent forms were obtained that had been approved by the Institutional Review Boards of Tulane Health Science Center, the University of Rochester School of Medicine, and the University of Mississippi Medical Center in Jackson. Myoblasts were propagated by collagenase and dispase dispersion of the tissue, establishment of the cell strain in F10 medium with 50% MRC-5 F10 conditioned medium, pre-plating to remove contaminating fibroblasts, growth in the presence of 10 ng/ml bFGF and 1 μM dexamethasone, and differentiation by limiting serum (2% horse serum for 1 day and 15% horse serum for 4-6 days), as described in more detail in Additional File 4. Each batch of cells was checked by immunocytochemistry for desmin (Thermo, RB-9014-P), a marker for muscle cells. The extent of myotube formation was determined by immunostaining for desmin and myosin heavy chain (MF20 monoclonal antibody from Stephen Hauschka) and determining the percentage of nuclei in multinucleated cells.

### Microarray expression analysis

Myoblast cultures at ~70% confluence and myotube preparations harvested 5 - 7 days after induction of differentiation were snap-frozen and stored in liquid nitrogen. Total RNA was isolated by standard methods (TRIzol extraction and RNeasy column, QIAGEN), that included DNaseI digestion. RNA was checked for quality (Nanodrop and Agilent Bioanalyzer), and 1 μg was labeled by a standard procedure that included a riboreduction step (Whole Transcript Sense Target labeling protocol, Affymetrix). The fragmented biotin-labeled cDNA was hybridized for 16 h to Affymetrix Exon 1.0 ST arrays and scanned (Scanner 3000 7 G, AGCC software, Affymetrix). After confirming the quality of the resulting .cel files (Affymetrix Expression Console software), they were imported into GeneSpring GX10. The data were quantile-normalized using only core-level transcripts (RMA) and baseline transformed to the median of all samples. The probe sets were further filtered to exclude ones that lie in the bottom 20th percentile across all samples. The raw data were deposited in the GEO database under series GSE26145.

### qRT-PCR

RNA was isolated from snap-frozen myoblasts and myotubes (RNeasy Mini kit; QIAGEN). After treatment of the RNA (RNA Quality Indicator ≥ 9; Experion, Bio-Rad) with DNaseI (Turbo DNA free, Ambion), cDNA was prepared using 1 μg of total RNA (Protoscript M-MuLV First Strand cDNA synthesis kit with Random Primer Mix; NEB). RT-PCR was performed with SYBR Green detection (RT² SYBR^® ^Green qPCR master mixes, SABiosciences; iCycler MyiQ, Bio-Rad). Unless otherwise noted, amplifications were done in duplicate using 20 - 40 ng of cDNA and 0.2 μM primer (Invitrogen) and the following parameters: 95°C, 30 s; annealing at an optimized temperature, 30 s; 72°C, 30 s for 45 cycles. Primers were designed http://frodo.wi.mit.edu/primer3/ and checked for the predicted specificity http://www.ncbi.nlm.nih.gov/tools/primer-blast/. Calibration curves for primer-pairs with serial 10-fold dilutions of a mixture of FSHD and control myotube and myoblast cDNAs gave slopes of -3.3 ± 0.4 and correlation coefficients of ≥ 0.98. Melting curves from PCR products were confirmed to give a single peak. The normalization standard for qRT-PCR was *M6PR*, which, according to the microarray data, had the following average expression ratios for FSHD vs. control myotubes and FSHD vs. control myoblasts: 0.97 (*p *= 0.9) and 1.27 (*p *= 0.2), respectively. *M6PR *was the most stably expressed gene from a set of seven standards (*M6PR, MSN, POLR1D, PPIA, B2M, EIF4A2*, and *MAT2A*) tested on FSHD vs. control myotube samples [35] http://medgen.ugent.be/~jvdesomp/genorm.

### Statistical analysis

ANOVA models for the comparison of gene expression levels across sample groups were fit using the limma package [36] in Bioconductor http://www.bioconductor.org, and heat maps were generated using the R gplots package http://CRAN.R-project.org/package=gplots. The Benjamini-Hochberg correction was used to adjust for multiple testing, and *p*-values of < 0.01 were considered significant. Chi-square tests for association and regression analyses were performed using R version 2.10 http://www.R-project.org.

## Results

### Cell samples for analysis

**Figure 1:**
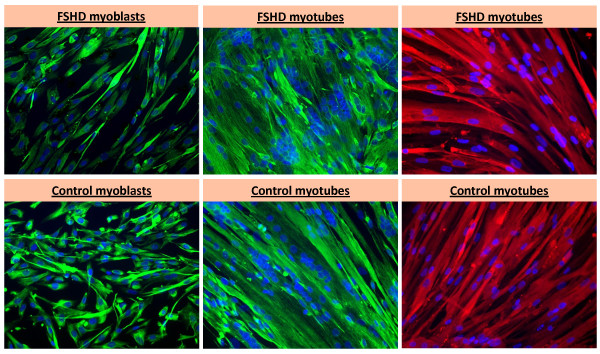
**Examples of myogenesis-specific immunostaining of FSHD and control myoblasts and myotubes used for the analysis**. Green, desmin; red, myosin heavy chain (MF20); blue, DAPI. The last panels in both rows were myotube preparations doubly stained with MF20 and DAPI. The occasional small bright green or red dots are a staining artifact. Fibroblasts do not stain with the desmin antibody nor with MF20.

FSHD myoblasts were generated from moderately affected muscle (See Additional File 1 for details about samples). The control myoblasts were from two normal individuals and one disease-control patient (sporadic inclusion body myositis). The myoblast preparations contained 90-98% desmin-positive cells before differentiation to myotubes. Therefore, there was minimal contamination with muscle-derived fibroblasts. The minor portion of fibroblasts may nonetheless have influenced the behaviour of the myoblasts by cell-signaling and might have made a contribution to the array signal, although only a minor one, as indicated by the consistent differences between FSHD and control samples. The myotube preparations had 72-80% of the nuclei in multinucleated myotubes (more than two nuclei per cell) after differentiation in comparison to the myoblast preparations with < 1% multinucleated cells (Figure 1). The growth medium for propagating myoblasts contained 10 ng/ml of basic fibroblast growth factor (bFGF) plus 1 μM dexamethasone, as recommended in studies of normal and FSHD myoblasts [37,38] and as used in recent studies of *DUX4 *expression [7,8]. For differentiation to myotubes, cells were incubated in the absence of bFGF and dexamethasone and with replacement of the normal 20% fetal bovine serum by 2% horse serum for one day, and then 15% horse serum for 4-6 days. Both the control and FSHD myotube preparations had cells with a more normal appearance and a higher percentage of myotubes due to the increase in the horse serum concentration after the first day of serum limitation. Dexamethasone improved the growth, the maximum number of cell population doublings, and appearance of control and FSHD myoblasts, as did regular additions of basic fibroblast growth factor. FSHD and control myoblasts and myotubes looked similar by phase microscopy (Figure 1), and the FSHD and control myoblasts grew and differentiated to myotubes equally well.

### Myogenesis-specific differences in gene expression

**Figure 2:**
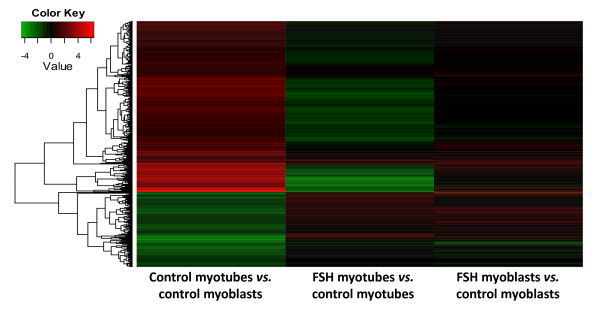
**Differentiation of control myoblasts to myotubes involved mostly increases in gene expression that were often diminished during FSHD myogenesis**. This heat map of log2 expression values (see color key) for genes upregulated > 2-fold in control myotubes vs. myoblasts shows that many genes that were upregulated during normal myogenesis (red in first column) were downregulated in FSHD vs. control myotubes (green in the corresponding portion of the second column). A small fraction of these were also downregulated in FSHD vs. control myoblasts (green in the corresponding portion of the third column).

We used an exon-based microarray (Affymetrix Exon 1.0 ST) to profile the expression of myoblast cultures harvested at ~70% confluence from FSHD and control myoblasts and from myotube preparations (three each). Upon differentiation of control myoblasts to myotubes, predominantly upregulation of gene expression was seen (Figure 2). Setting the fold change (FC) threshold for control myotubes vs. control myoblasts of > 2.0 at a significance level of *p *< 0.01, 511 genes were upregulated and 224 downregulated; all *p*-values for array data were adjusted for multiple comparisons using the Benjamini-Hochberg correction. As expected, the group of genes that was upregulated in control myotubes vs. myoblasts contained a very strong overrepresentation of muscle genes. Seventeen out of 20 of the most overrepresented GO terms for this set of genes were specifically related to muscle and the other three were actomyosin structure organization, cell adhesion, and biological adhesion (DAVID functional analysis tool). The analogous downregulated group of genes had only several strong associations with GO terms, and all involved the plasma membrane.

**Figure 3:**
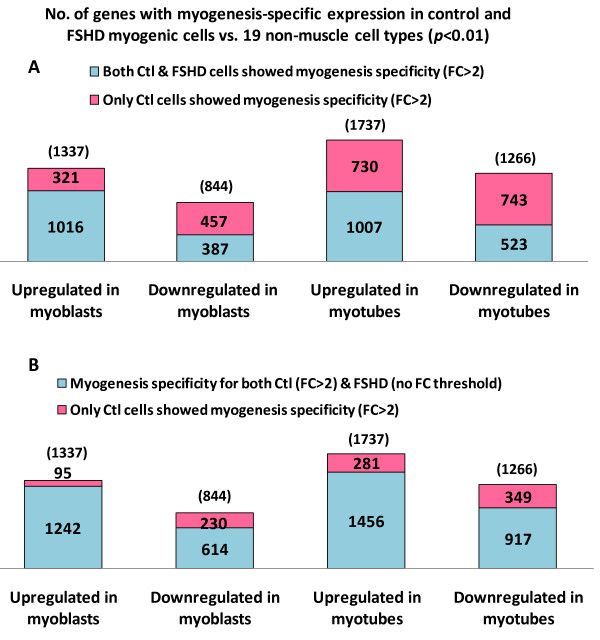
**The number of genes with myogenesis-specific expression: comparison of control and FSHD cells with 19 non-muscle cell types**. **A**. The number of genes with FC > 2 (*p *< 0.01) in comparison of both control and FSHD cells with 19 non-muscle cell types. The total number of genes with FC > 2 (*p *< 0.01) for upregulation or downregulation in control myoblasts or myotubes vs. 19 diverse non-muscle cell cultures is given in parentheses. The divisions in the bars show the overlap of the set of myogenesis-specific genes in FSHD myoblasts or myotubes with that of the corresponding control myogenic cells. **B**. The number of genes with overlapping myogenesis specificity for FSHD and control myoblasts and myotubes but relaxing the stringency for the FSHD myogenic cells to only significant myogenesis-specific differences (*p *< 0.01) without an FC specified while retaining the FC > 2 threshold for control myogenic cells.

Also available for determination of myogenesis-specific gene expression was the powerful resource of our expression profiling of 19 heterologous non-muscle cell types as part of the ENCODE project (See Additional File 2 for descriptions of the cell types; Crawford GE, unpub. data). The diverse non-muscle cell cultures, which included fibroblasts, melanocytes, hepatocytes, and astrocytes, were profiled using the same type of exon microarrays and identical methods as for the myoblasts and myotubes. An analysis of variance (ANOVA) model was fit to determine expression differences between myoblasts or myotubes and these non-muscle cell types. The majority of the gene expression differences (FC > 2, *p *< 0.01) between control myoblasts and non-muscle cells involved increases in expression (Figure 3A). The 20 GO terms most overrepresented among the myoblast-upregulated genes were associated with the plasma membrane, muscle, the actin cytoskeleton, cell adhesion, and enzyme-linked receptor protein signaling; also prominent in this set of genes were GO terms for extracellular structure organization, calcium ion binding, response to oxygen levels, and GTPase regulator activity. The GO terms associated with the group of myoblast-downregulated genes were more diverse but featured sterol metabolism, cell cycling, and cell-cell junction. As expected, among the genes upregulated in control myotubes vs. non-muscle cell types, muscle GO terms predominated but extracellular structure organization, cell adhesion, actin cytoskeleton, transmembrane receptor, and GTPase regulator activity were also prominent. For genes downregulated in control myotubes vs. non-muscle cell types (Figure 3A), almost all the top 20 overrepresented GO terms were related to cell division.

### Extensive dysregulation of gene expression in FSHD myoblasts and myotubes

Comparing FSHD and control myoblasts, 1.7% of the ~17,000 genes represented on the microarray displayed FC > 2.0 in expression at a significance level of *p *< 0.01. Remarkably, only 10% of these 295 FSHD myoblast-dysregulated genes were downregulated (Figure 2). At the myotube stage there were almost three times as many FSHD-dysregulated genes (797 genes; 4.7% of the genes on the microarray) and a much higher percentage was downregulated (37%). FSHD dysregulation at the myoblast stage was significantly associated with dysregulation at the myotube stage. For example, in FSHD-to-control comparisons, 266 and 502 genes were upregulated more than 2-fold in myoblasts and myotubes, respectively, and 130 genes were found in both sets of FSHD-upregulated genes (*p *< 0.00001, Chi-square test). Therefore, 49% of the genes upregulated with a FC > 2 and *p *< 0.01 in FSHD vs. control myoblast samples (3 each) were similarly upregulated in FSHD vs. control myotube samples (3 each). Similarly 39% of the 29 genes downregulated more than 2-fold in FSHD vs. control myoblasts were also downregulated more than 2-fold in FSHD vs. control myotubes. It was not expected that 100% of the genes would behave similarly because of the extensive differences in control myoblast-specific vs. control myotube-specific transcription described above.

Previously reported evidence suggested that 4q35.2 genes *FRG1*, 4q35.1 gene *ANT1 (SLC25A4)*, and the 5q31 gene *PITX1*, which were represented on our microarrays, might be involved in FSHD [6,17-22]. In accord with other qRT-PCR studies [23,24,39], we did not observe dysregulation of *FRG1 *or *PITX1*. Although detection of transcripts from *FRG1 *is complicated by its cross-homology to many transcribed genomic sequences, even in the most specific probe-sets for the gene located in the first exon, similar signal intensities were seen for FSHD and control myogenic cells. We did observe that *ANT1 *was upregulated 2-fold in FSHD vs. control myoblasts (*p *= 0.006), although not in myotubes. Other 4q35.2 genes represented on the microarray were *FAT1 *(the only 4q35.2 gene with muscle-lineage specific DNaseI hypersensitivity sites [24]), *F11, CYP4V2, MTNR1A, ZFP42, TRIML1*, and *TRIML2*. They showed no differences in RNA signal between FSHD and control myogenic cells. In addition, the muscular atrophy-associated gene *FBXO32 (*atrogin-1) showed no significant change in FSHD vs. control myogenic cells. Another atrophy-associated gene *TRIM63 (MURF1) *had decreased, not increased, RNA signal in FSHD myotubes vs. control myotubes (FC = -2.3, *p *< 0.0001). No significant FSHD-related changes were seen for the muscle-differentiation inhibitory *MSTN (*myostatin)*, ID1, ID2*, or *ID3 *genes.

**Figure 4:**
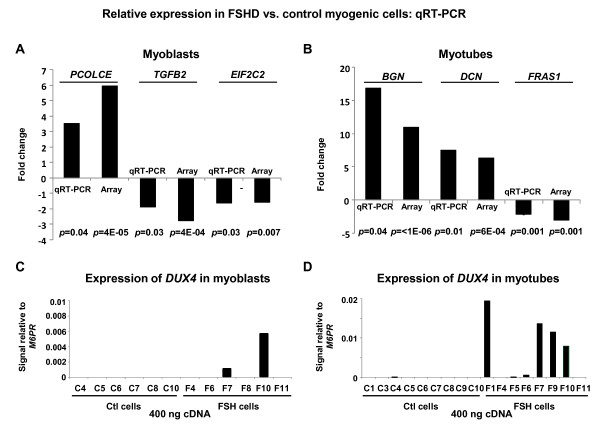
**Examples of qRT-PCR validation of microarray expression data and *DUX4 *expression by RT-PCR**. **A**. and **B**. By qRT-PCR on 20 - 40 ng of cDNA template, the relative expression of representative genes in FSHD vs. control myoblast or myotube samples from different individuals was determined for representative genes that displayed significant FSHD-linked differences on the microarray (Table 1). qRT-PCR signals for the test amplicons were normalized to those of *M6PR*. The average fold-changes deduced from the qRT-PCR and the microarray analysis are shown, and the *p*-values for the FSHD/control differences are indicated. **C**. and **D**. To detect *DUX4-fl *RNA (fl, full-length isoform, associated with FSHD), 400 ng of cDNA was used for real-time PCR as previously reported [7]; 20 - 40 ng of cDNA was used for the other amplicons. Results from different individual FSHD and control cell strains are shown. The samples used for *DUX4 *RT-PCR were the same as those used for qRT-PCR validation of microarray results.

By qRT-PCR, using mostly cDNAs not analyzed on the microarray (Table 1 Figure 4A and 4B, Additional File 3 for sources of cDNA, and Additional File 5 for primers), we validated twelve sets of microarray-determined FSHD-associated differences in expression. Some of these genes were part of functionally related sets that showed similar FSHD-related changes in expression in the microarray data. For example, we quantified *MYOM1 *expression by qRT-PCR (Table 1), and, according to the microarray data, the muscle-associated myomesin genes, *MYOM1, MYOM2*, and *MYOM3 *were all downregulated in expression in FSHD vs. control myotubes (FC = - 8.9, -8.1, and -3.8, respectively, each *p *< 0.0005). Nonetheless, these genes still had significantly more expression in FSHD myotubes than in the non-muscle cell samples (FC = 3.2, 1.5, and 3.8, respectively, each *p *< 0.0001).

**Table 1:** Validation by qRT-PCR of FSHD dysregulation of gene expression

	Microarray profiling		qRT-PCR			
			
Gene	FSH/Ctl FC^b^	*p*-value	FSH/Ctl FC^b^	*p*-value	No. of FSH samples	No. of Ctl samples
**Myoblasts**						

*PCOLCE*^a^	5.9	4E-05	3.5	0.04	5	6
*RUVBL2*	2.2	5E-04	1.7	0.01	7	8
*TGFB2*	-2.8	4E-04	-1.9	0.02	9	8
*EIF2C2*	-1.6	0.007	-1.6	0.03	8	8
**Myotubes**						

*BGN*	11.0	< 1E-06	16.8	0.04	8	9
*DCN*	6.4	6E-04	7.5	0.01	8	9
*PCOLCE*	10.2	< 1E-06	3.7	0.03	6	7
*MYOM1*	-8.9	2E-06	-2.9	0.05	4	4
*SFRS9*	3.5	< 1E-06	3.5	0.01	6	6
*CAPG*	3.0	0.002	3.2	0.02	8	9
*IGFBP6*	2.5	0.02	3.5	0.01	8	9
*FRAS1*	-3.02	0.001	-2.2	0.001	4	4

^a^Full gene names and primer-pairs are given in Additional File 5 TableS4

^b^FC, fold change in RNA signal was determined by exon microarray analysis or qRT-PCR

The gene chosen for normalization of the qRT-PCR data was *M6PR *on the basis of its essentially identical average expression levels in control and FSHD myoblasts and myotubes, as determined from the microarray expression data. The often-used *GAPDH, PPIA*, and *B2M *genes were not optimal standards because of difficulty in finding unique primer-pairs due to closely related genes or pseudogenes elsewhere in the genome (*GAPDH *and *PPIA*) or FSHD-linked differences in expression levels (*B2M *and several *GAPDH*-related glycolytic enzymes). Our finding that ~800 genes showed more than 2-fold differences in expression in FSHD vs. control myotubes (*p *< 0.01) and many more showed significant differences that were less than 2-fold indicates the importance of using expression profiling data to choose gene standards for qRT-PCR that will not give artifacts in determining relative expression levels of test genes.

We also assayed relative levels of *DUX4-fl *RNA, the RNA isoform associated with FSHD, using very high amounts of cDNA as template, namely, 400 ng, as per the method of Lemmers et al. [7]. We could detect *DUX4-fl *RNA in some of the FSHD myotube and myoblast samples (Figure 4C and 4D). None of the control myoblasts and myotubes gave appreciable signal. The Ct values from positive FSHD myoblasts and myotubes were usually much higher, namely, 33-36, than for the other tested amplicons with the same cDNAs despite the use of 10-20 times more cDNA for the *DUX4 *RT-PCR. We do not have microarray results for comparison to the *DUX4 *RT-PCR data because *DUX4 *is not represented on the microarray due to its extensive cross-hybridization in the genome [40].

### Dampening of muscle-specific transcription changes in FSHD vs. control myogenic cells

**Figure 5:**
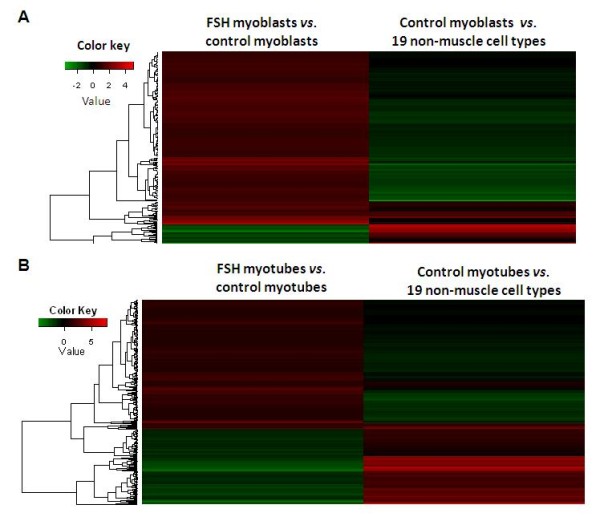
**Inverse relationships between myoblast- and myotube-specific gene expression changes and FSHD-specific changes**. **A**. Genes up- or downregulated in FSHD vs. control myoblasts often showed opposite changes in expression in control myoblasts vs. 19 non-muscle cell types. This heat map of log2 expression ratios for genes upregulated > 2-fold (*p *< 0.01) in FSHD vs. control myoblasts illustrates that these genes were often downregulated in control myoblasts vs. diverse non-muscle cell cultures and that there was the converse relationship for genes downregulated in FSHD vs. control myoblasts and upregulated in control myoblasts vs. non-muscle cell cultures. Both associations were significant (*p *< 0.01). **B**. This heat map is analogous to that of Panel **A **except that it is for myotubes instead of myoblasts.

**Figure 6:**
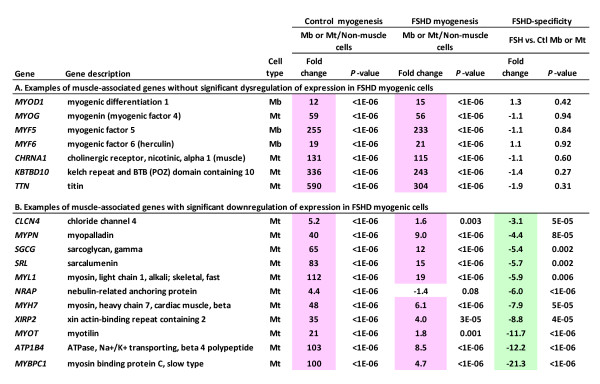
**Some muscle-associated genes are downregulated in FSHD myogenic cells while others are not**. Fold changes in RNA signal were determined by identical expression profiling of FSHD and control myoblast and myotube preparations and 19 diverse non-muscle cell types (See Additional File 1 and Additional File 2 for detailed descriptions of samples). All *p*-values for microarray data were adjusted for multiple comparisons; pink, significantly upregulated at *p *< 0.01; green, significantly downregulated at *p *< 0.01. Myotube (Mt) data are shown for the genes with much stronger expression at the myotube stage than at the myoblast stage. Myoblast data are given for *MYOD1, MYF5*, and *MYF6*.

Downregulated expression in FSHD vs. control myogenic cells was associated with upregulated expression in control myogenic cells vs. non-muscle cell types (*p *< 0.01; Figure 5). The analogous inverse relationship was seen for genes upregulated in FSHD vs. control myogenic cells. Often, the downregulation in FSHD myoblasts or myotubes relative to the analogous control cells was a dampening of myogenesis-linked expression increases, rather than the absence of these normal myogenesis-related expression changes (See Additional File 6 and Additional File 7 for fitted regression analysis). This dampening of myogenesis-specific expression is evidenced by the finding that a much higher percentage of genes showed myogenesis specificity in FSHD myogenic cells vs. non-muscle cell types when the expression differences had to meet the criterion of *p *< 0.01 but no fold-change threshold of 2.0 was set as for the control myogenic cells (Figure 3B vs. 3A). Examples of genes with dampening of myogenesis-specific expression changes in FSHD cells are shown in Figure 6B. However, other myogenesis-associated genes, e.g., the critical myogenesis-specific transcription factors *MYOD1 *and *MYOG*, displayed no change in RNA levels in FSHD vs. control myoblasts or myotubes (Figure 6A). Some genes were dysregulated in FSHD myoblasts or myotubes but did not exhibit inverse expression changes in myogenic vs. non-muscle cells. For example, 5% of the genes upregulated in FSHD vs. control myotubes (FC > 2, *p *< 0.01) were also upregulated in control (and FSHD) myotubes vs. non-muscle cell types. In this group there was an overrepresentation of genes associated with inflammation or encoding extracellular proteins.

### Transcriptional dysregulation in FSHD

**Figure 7:**
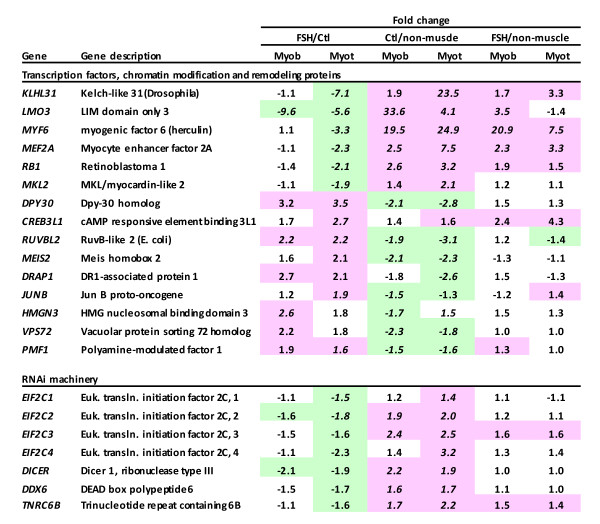
**Transcription control and RNAi machinery genes dysregulated in FSHD cells**. Pink or green highlighting indicates significant up- or downregulation, respectively, for the indicated comparison at *p *< 0.01; italics, *p *< 0.001. The FSHD/Ctl difference in expression for *MEIS2 *was the exception with *p *= 0.02. *KLHL31, RUVBL2*, and *EIF2C2 *had been tested and validated for FSHD-associated dysregulation by qRT-PCR (Table 1).

Some transcription regulatory genes were significantly dysregulated in FSHD vs. control myogenic cells (Figure 7). *MEF2A, RB1, MKL2*, and *KLHL31*, which are associated with transcription control during myogenesis [41,42], were downregulated in FSHD vs. control myotubes. FSHD myotubes relative to those of controls displayed upregulation of *JUNB *and *CREB3L1*, which are transcription factors with protective roles for muscle or cellular stress [43,44]. In FSHD vs. control myoblasts, *DPY30, RUVBL2, DRAP1, PMF1, HMGN3*, and *LMO3 *were among the genes involved in the control of transcription that were significantly dysregulated.

To obtain additional evidence for dysregulation of steady-state RNA levels in FSHD being governed, in part, by differential activity of transcription factors, we analyzed the distribution of predicted transcription factor binding sites (TFBS) among the promoter regions of a set of 826 genes. These were approximately equally divided between genes that were upregulated and those that were downregulated in FSHD vs. control myotubes. Four of the 126 analyzed TFBS motifs were significantly skewed toward either FSHD up- or downregulation *(p *< 0.01; Table 2). MEF2A and E4BP4 DNA-binding motifs were enriched among FSHD-downregulated genes, which is consistent with the downregulation of *MEF2A *RNA in FSHD vs. control myotubes (Figure 7) and its role in myogenesis and the role of E4BP4/NFIL3 as a transcription repressor. The motifs for TP53, which is associated with inflammation, and PPARG, which is associated with stress response and lipid metabolism, were significantly correlated with genes that were upregulated in FSHD vs. control myotubes. There was no change in *TP53 *RNA levels and only a ~1.5-fold increase (*p *= 0.02) in *PPARG *RNA levels; however, expression of the protein products, their modification, and interactions might have changed in FSHD cells.

**Table 2:** TFBS motifs associated preferentially with FSHD up- vs. downregulated genes in myotubes.

	**No. of genes with the motif from 826 promoter regions**^**a**^			*p*-value: skewing of up- or dnreg.	**Prominent functional annotation**^**b**^	
			
TFBS motif	**FSH upreg**.	**FSH dnreg**.	**Upreg./dnreg**.		**FSH upreg**.	**FSH dnreg**.
MEF2A	39	101	0.4	< 0.0001	PI3K signaling, 8 genes	muscle phenotype, 16 genes
E4BP4	16	42	0.4	0.008	cell adhesion, 5 genes, GO:0007155	none
PPARG	100	43	2.3	0.0001	extracellular matrix, 13 genes, GO:0031012; arachidonic acid, 15 genes	cardiac hypertrophy, 7 genes
p53	84	37	2.3	0.0009	inflammation, 29 genes; superoxide, 29 genes	Activation of cAMP-dependent PKA, 5 genes

^a^Predicted transcription factor binding site motifs (TFBS) in the upstream (promoter) region for 403 and 423 genes that displayed the strongest up- or downregulation (p < 0.01), respectively, in FSHD vs. control myotubes. These sites were identified by GeneCards http://genecards.org and were analyzed by Chi-square tests for association with FSHD up- vs. downregulation in myotubes. Of the 126 TFBS scored, the four shown in this table had the strongest association with either FSHD up- or downregulated genes. For PPARG, PPARG1 and PPARG2 motifs were pooled.

^b^Functional annotation from GeneDecks http://genecards.org

### Evidence for post-transcriptional dysregulation of RNA levels in FSHD

Genes encoding the components of the RNA-induced silencing (RNAi) machinery, including all four Argonaute genes, were significantly downregulated in FSHD *vs*. control myotubes and two of them were also downregulated in FSHD *vs*. control myoblasts (Figure 7). The downregulation of *EIF2C2 *(Argonaute 2) in FSHD myoblasts was confirmed by qRT-PCR (Figure 4A). Surprisingly, these genes showed significantly higher expression in control myogenic cells vs. 19 non-muscle cell types (Figure 7). In FSHD myogenic cells compared with the non-muscle cell types, these genes were not downregulated; most of them were just not upregulated.

Exon-based microarrays can reveal the presence of cell type-specific differences in RNA isoforms from a given gene by analysis of exon-normalized probe data instead of the gene-normalized data described above. By gel electrophoresis, we examined oligo(dT)_23_-primed RT-PCR products (0.2 - 0.5 kb) from five of the genes for which the exon-normalized array data suggested FSHD-specific RNA isoforms. None of these representative genes (*FAT1, SCUBE3, ILF3, TFPI2*, or *SFRS7*) was validated as giving the predicted FSHD-specific differences in RNA sizes. In addition, we looked for previously reported FSHD-associated RNA isoforms from *FXR1P, TNNT3*, and *MTMR1 *[17,45] but did not see any evidence for them in our cell populations.

### Functional terms associated with genes dysregulated in FSHD myoblasts and myotubes

A bioinformatics analysis was done to look for functional terms associated with genes significantly dysregulated in FSHD myoblasts or myotubes (*p *< 0.01). Functional terms overrepresented among FSHD-upregulated genes (e.g., mitochondrial terms, extracellular matrix, Rho) were mostly very different from those for FSHD-downregulated genes (e.g., myofibril, RNA-induced silencing complex; Table 3). Many of the genes that were upregulated in both FSHD myoblasts and myotubes were related to the response to cell stress, such as *GSTP1, HSP90AA1 HSP90AB1 HSPA1A, HSPC152*, and *DNAJC4 *(See Additional File 8 for gene lists). However for cell stress genes, as for most of the overrepresented functional terms among FSHD-dysregulated genes, the up- or downregulation in FSHD vs. control myogenic cells was often associated with down- or upregulation, respectively, in control myogenic cells vs. non-muscle cells. This is illustrated in Additional File 9 for FSHD-dysregulated pro- and anti-apoptosis genes.

**Table 3:** Some pathways and functional terms overrepresented among FSHD-dysregulated genes.

**Functional terms**^**a**^	Mb or Mt	No. of up- or dnreg. genes	**Ratio**^**b**^
RNA-induced silencing complex	Mt	4 dn	0.80
	Mb	1 dn	0.20

Fatty acid elongation in mitochondria	Mb	7 up	0.44
	Mt	2 up	0.13

Extracellular matrix	Mt	35 up, 1 dn	0.30
	Mb	4 up, 2 dn	0.05

NRF2-mediated oxidative stress response	Mt	15 up, 7 dn	0.13
	Mb	7 up	0.04

HIF1α Signaling	Mt	6 up, 7 dn	0.12
	Mb	5 up	0.05

Regulation of actin-based motility by Rho	Mt	9 up, 3 dn	0.10
	Mb	6 up	0.07

Mitochondrial matrix	Mt	18 up, 5 dn	0.11
	Mb	22 up, 1 dn	0.11

Oxidative phosphorylation	Mt	18 up	0.16
	Mb	11 up	0.09

Induction of apoptosis	Mt	13 up, 5 dn	0.09
	Mb	8 up, 2 dn	0.05

Anti-apoptosis	Mt	16 up, 3 dn	0.10
	Mb	4 up	0.02

^a^Overrepresented functional terms for genes up- or downregulated (p < 0.01) in FSHD vs. control myoblasts (Mb) or myotubes (Mt) were identified by bioinformatics programs. Details and the names of the dysregulated genes are given in Additional File 8 Table S5.

^b^The number of genes for a given pathway or GO-term that were up- or downregulated in FSHD vs. control myogenic cells/the total number of genes for that pathway or GO-term that were included in the microarray.

## Discussion

**Figure 8:**
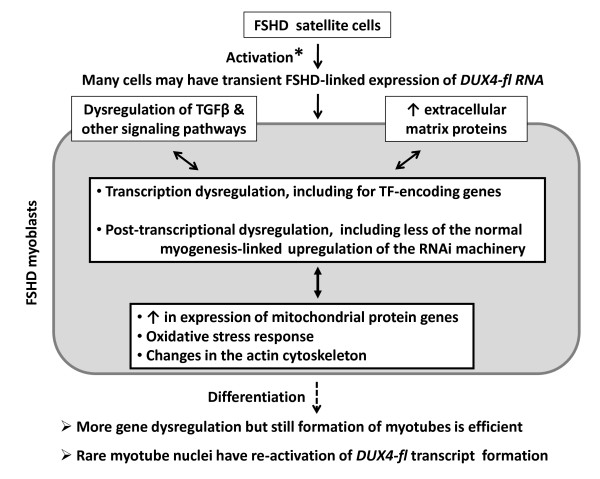
**Proposed scheme for FSHD pathogenesis in myoblasts**. This cartoon shows some of the main regulatory changes at the myoblast stage that may be determining the FSHD-specific gene expression profile in myoblasts and myotubes. *DUX4-fl *RNA is found at extremely low levels in FSHD and control myoblasts and myotubes but not in the corresponding control cells. The double-headed arrows indicate that these regulatory changes can reinforce each other. TF, transcription factors. RNAi, RNA interference; *, the activation of *DUX4-fl *expression in FSHD at the satellite cell stage is proposed to occur at or before activation of FSHD satellite cells and is not yet tested.

Our study is the first to examine in parallel many different human cell types and compare them to well-characterized FSHD and control myoblasts and myotubes and thereby demonstrate extensive FSHD-linked dysregulation of gene expression. Importantly, we confirmed by RT-PCR that the disease-associated RNA isoform of *DUX4, DUX4-fl *RNA, is expressed at extraordinarily low levels in FSHD (but not detectable in control) myoblasts and myotubes despite the hundreds of genes dysregulated more than 2-fold in FSHD vs. control myoblasts and myotubes. Our findings of extremely infrequent expression of *DUX4-fl *RNA in FSHD myoblasts and myotubes and undetectable levels in some of these FSHD cell populations are consistent with previous reports. This transcript was detected in only ~1 out of 1000 FSHD myotube nuclei and was observed less frequently in FSHD myoblasts than in myotubes [8]. Similarly, its detection by one round of real-time PCR required much higher-than-normal amounts of FSHD myotube cDNA [7]. Because FSHD myoblasts and myotubes had a strong transcription dysregulation profile (this study) and FSHD myoblasts are hypersensitive to oxidative stress [14,15], if *DUX4 *is the first pathologically dysregulated gene in FSHD, then it must be expressed much more extensively, but transiently, at an earlier stage in myogenesis. We propose that *DUX4-fl *RNA initiates a cascade of gene dysregulation at or before activation of FSHD satellite cells to form myoblasts (Figure 8). *DUX4-fl *transcripts in myotubes and myoblasts would then represent a rare re-activation of inappropriate *DUX4 *expression that is not central to pathogenesis. This model contrasts with the current emphasis on the rare expression of *DUX4-fl *RNA in myoblasts and myotubes.

Induction of *DUX4-fl *transcription in transduced C2C12 myoblasts caused apoptosis; inhibited differentiation to myotubes; gave dramatic changes in cell shape; and, even at sublethal concentrations, inhibited transcription of *MYOD1 *and *MYOG *and increased that of *CDKN1A *(p21) [27]. In addition, *DUX4-fl *RNA injected into Xenopus or zebrafish embryos is highly cytotoxic [21,46]. In contrast, we found only minimal apoptosis in FSHD myoblasts and myotubes and no more than in controls (~5% of nuclei as detected by staining with ethidium bromide and acridine orange [47] and no internucleosomal fragmentation of DNA [48], data not shown). In our comparison of FSHD and control myotubes, more anti-apoptotic than pro-apoptotic changes in gene expression were seen and the dysregulation of most of these genes can be explained by FSHD-associated changes in the normal myogenesis program (See Additional File 9). The good growth and efficient differentiation to myotubes of FSHD myoblasts observed by us and others [15] are consistent with our finding that the myogenic regulatory factors *MYOD1, MYOG, MYF5*, and *MYF6 *were equally highly expressed at the RNA level in FSHD vs. control myoblasts and only *MYF6 *was significantly downregulated in FSHD vs. control myotubes. This result argues against FSHD-related differences in posttranscriptional processing of the products of these four genes in myoblasts because their expression is autoregulatory [49-52]. In addition, we observed no differences between FSHD and control myogenic cells in RNA levels for E-box protein heterodimer partners of these myogenic regulatory factors (data not shown). In support of our model of the proposed non-cytotoxic expression of *DUX4 *very early in myogenesis (Figure 8), the early-myogenesis transcription factors *PAX3 *and *PAX7 *can partly counteract the deleterious effects of overexpression of *DUX4 *in C2C12 cells [27]. Moreover, *DUX4-fl *transcripts are normally rather abundant in testis [8].

Previously used arrays for transcription profiling of well-characterized FSHD and control myogenic samples [10-12,14] did not have multiple probe-sets per exon for each transcript nor probes for exons other than the 3' exon and so are much less representative of the transcript populations. Given this major difference and the use of muscle tissue rather than myoblasts (a very minor component of muscle) in most of the previously published FSHD RNA profiling studies, it is not surprising that there was only minimal overlap between genes reported as dysregulated in FSHD in previous studies and genes that we observed to be dysregulated in FSHD vs. control myogenic precursors. An example of such infrequent overlap is the upregulation of the vascular smooth muscle-associated *CTGF, ENG*, and *TAGLN *genes in FSHD vs. control muscle [12] and, in this study, in FSHD vs. control myotubes (2- to 3-fold upregulation, *p *< 0.001 for all comparisons). In the previous expression profiling of well-characterized FSHD and control myoblasts, a "vacuolar/necrotic phenotype" was noted for "the majority" of FSHD myoblasts, which were "markedly swollen." That morphological phenotype might be due to FSHD myoblasts being more sensitive to stress than analogous controls. Because we used only moderately affected muscle to generate myoblast cell strains and FSHD is characteristically a slowly progressive disease in which disease muscle biopsies look relatively normal at the time of clinical onset [53], the normal appearance of FSHD myoblasts and myotubes under our optimized growth conditions is likely to be relevant to understanding pathogenesis. Moreover, the equally good generation, propagation, growth, and differentiation of FSHD and control myoblasts also argue against the possibility that we selected a non-representative sub-phenotype of FSHD myoblasts. Similarly, these observations fit the high degree of correlation of the overall expression profiles of all the FSHD and control myogenic samples with each other when compared to the 19 non-muscle cell populations despite the hundreds of significant more-than-two-fold differences in RNA signal for individual genes in comparisons of FSHD and control myoblasts or myotubes.

There was a recent report by Cheli et al. [16] about exon array-based expression profiling of FSHD and control myoblasts and myotubes but it included no characterization of the percentage myoblasts in the studied cell populations nor the efficiency of differentiation to myotubes. The extent of contamination of untransformed myoblast cultures with non-muscle cells can vary dramatically between different myoblast cell strains and even at different passage numbers and, thereby, have a major impact on expression profiling. Cheli et al. reported < 4% overlap between several hundred genes with dysregulation in FSHD vs. control cells at the myoblast stage and those dysregulated at the myotube stage, unlike the present study in which we found 48% overlap between genes with FSHD dysregulation (*p *< 0.01, FC > 2) in myoblasts and myotubes. The difference in the results from the study of Cheli et al. and the present study might be due to the unknown percentage of cells that differentiated in their experiment vs. 72-80% in ours.

Our analysis of normal myogenesis from a comparison of expression profiles of control myoblasts and myotubes and 19 non-muscle cell types indicated the prominent role of upregulation of genes generally involved in the actin cytoskeleton, organization of the extracellular matrix, cell adhesion, and GTPase regulator activity, in addition to the expected muscle-specific genes. One unexpected functional class of genes that was more highly expressed in control myoblasts and myotubes than in non-muscle cells was the RNA silencing machinery genes. These same RNA silencing machinery genes were lacking upregulation during FSHD myogenesis, which may contribute to the observed excess of FSHD-upregulated vs. FSHD-downregulated genes in myogenic precursors.

Our expression profiling of FSHD vs. control myoblasts suggests an explanation for the FSHD myoblasts' hypersensitivity to external oxidative stress [14,15] as well as an imbalance in the redox system in muscle [54]. The observed FSHD-related upregulation of many transcripts from oxidative phosphorylation genes could result in an increase in endogenous reactive oxygen species and might eventually result in apoptosis in some severely affected muscles. Accordingly, increases in H_2_O_2 _were seen in FSHD vs. control muscle [54] and upregulation of some mitochondrial oxidative phosphorylation proteins and oxidative stress-response proteins was observed in affected and also in unaffected FSHD skeletal muscle vs. normal-control muscle [20]. Similarly, we found FSHD-associated upregulation of RNA for oxidative stress-response and oxidative phosphorylation proteins, including several of the same proteins (*SOD1 *and *HSPB1*) whose FSHD-upregulation was seen in muscle [20]. The hypothesized inappropriate expression of *DUX4 *very early during regenerative myogenesis would help explain why even unaffected muscle showed these abnormalities in protein levels [20] and why myoblasts from unaffected FSHD muscle samples displayed an FSHD-associated hypersensitivity to oxidative stress [15]. Moreover, it would be consistent with our finding that myoblast cell strains from diverse, moderately affected FSHD muscle samples displayed FSHD-related changes in gene expression.

The most prominent feature of the transcription dysregulation in FSHD myoblasts and myotubes was the decrease in the up- and downregulation of RNA levels associated with normal myogenesis, which can account for most of the FSHD-related dysregulation. Expression profiling of other muscular dystrophies [55-58] has not revealed such a widespread dampening of normal myogenesis-associated transcription changes in various functional gene categories. Some classes of genes, including those encoding extracellular matrix or pro-inflammatory proteins, were strongly enriched in FSHD-upregulation in myogenic cells independent of any inverse myogenesis association. The proliferation of FSHD myoblasts and their differentiation to myotubes in vitro was unaffected by these changes in gene expression. Apparently, there is also not a large disease-related depletion of satellite cells in FSHD patients because of the above-mentioned finding that generating myoblast cell strains from moderately affected muscle biopsies of FSHD patients was no more difficult than from control muscle. Moreover, although Reed et al. [59] observed abnormal spatial relationships of the sarcolemma with the underlying contractile apparatus in affected FSHD muscle, the structure of the contractile apparatus itself appeared normal. The observed FSHD-associated gene dysregulation may have been heightened in the FSHD myoblasts and myotubes relative to their in-vivo counterparts due to the effects of cell culture and the use of the myoblast-stimulatory [37,38] dexamethasone in the culture medium for both FSHD and control myoblasts [60,61]. If extensive cell culture promoted increased gene dysregulation in FSHD myoblasts, this could be relevant to the disease in vivo because usually it is only slowly progressive. In addition, glucocorticoids are relevant in vivo because of the effects of endogenous glucocorticoids in traumatic or muscle wasting conditions [62,63] and the therapeutic use of glucocorticoids. Moreover, if only a small fraction of the extent of gene dysregulation that we saw exists in vivo, this could lead to atrophy by interfering with effective regenerative myogenesis. For example, all three of the skeletal muscle-associated myomesin genes showed downregulation in FSHD vs. control myotubes of about 4 to 9 fold, and the products of these genes bind to other muscle structural proteins in a dose-dependent manner as major components of the myofibrillar M-band.

It is not yet clear whether the dysregulated gene expression in FSHD myoblasts is due to disease-related differences in transcription regulatory or RNA-processing proteins, cell signaling (e.g., TGFβ or RHO/mTOR pathways [64,65]), indirect effects on transcription from overexpression of extracellular proteins [66], indirect effects of mitochondrial dysfunction [67], subtle differences in timing of expression of some myogenesis-specific gene(s), and/or disease-specific epigenetic differences. At the myotube stage, the increase in the number of genes that were dysregulated in FSHD cells may be partly due to the FSHD-associated decreases in expression of the transcription regulatory *MYF6 *and *MEF2A *genes and abnormal increases in expression of *MEIS2 *after induction of differentiation to myotubes.

## Conclusions

Given the extremely low rate of FSHD-associated inappropriate expression of *DUX4 *at the myoblast, myotube, and muscle stages, many of the FSHD-dysregulated transcription-regulatory or cell signaling genes revealed by our expression profiling may be more effective targets for developing pharmacologically-based or gene therapy-based treatment of FSHD than *DUX4 *itself. Our findings point to FSHD being a differentiation-associated disease, and so study of this enigmatic muscular dystrophy is likely to elucidate new aspects of normal myogenesis as well as pathogenesis. In addition, our comparison of transcription profiles of control myoblasts and myotubes and those of 19 other cell types that were examined identically showed how very extensive gene expression changes are upon formation of myoblasts and upon their differentiation to myotubes.

## Competing interests

The authors declare that they have no competing interests.

## Authors' contributions

KT prepared and characterized the myoblasts and myotubes and prepared the RNA and cDNA for qRT-PCR. S-CC did the qRT-PCR analyses. SVC did the hybridization and generated the initial data sets from the microarrays. ML did the statistical analyses of the expression profiling data. CB and ME did the analyses of functional categories of dysregulated genes. RB obtained the muscle biopsies for most of the myoblast cell cultures and JS generated most of the myoblast cell strains from them. GEC and SVC did the expression profiling of the 19 non-muscle cell types. ME wrote the MS. All authors read and approved the final manuscript.

## Pre-publication history

The pre-publication history for this paper can be accessed here:

http://www.biomedcentral.com/1755-8794/4/67/prepub

## Supplementary Material

Additional file 1 **Table S1**. Description of myoblast and myotube samples for microarray analysis Click here for file

Additional file 2 **Table S2**. Descriptions of non-muscle cell types for expression profiling Click here for file

Additional file 3 **Table S3**. Description of myoblast and myotube samples for qRT-PCR Click here for file

Additional file 4 **Additional Methods: Generation, propagation, and differentiation of myoblast cultures from human skeletal muscle**. Click here for file

Additional file 5 **Table S4**. qPCR primers and full names of genes tested. Click here for file

Additional file 6 **Figure S1**. FSHD-downregulated genes: relationship between gene expression in FSHD myotubes vs. non-muscle cell types to that in control myotubes vs. non-muscle cell types. Click here for file

Additional file 7 **Figure S2**. FSHD-upregulated genes: relationship between gene expression in FSHD myotubes vs. non-muscle cell types to that in control myotubes vs. non-muscle cell types. Click here for file

Additional file 8 **Table S5**. Some pathways and functional terms overrepresented among FSHD-dysregulated genes. Click here for file

Additional file 9 **Table S6**. Most of the pro- or anti-apoptotic genes that were dysregulated in FSHD vs. control myotubes were apparently up- or downregulated in FSHD myotubes because of dampening of their normal myogenesis-associated changes in expression. Click here for file
