# Supplementary material for: Gene expression during normal and FSHD myogenesis
Source: BMC Med Genomics. 2011 Sep 27;4:67. doi: 10.1186/1755-8794-4-67 (PMC3204225; doi:10.1186/1755-8794-4-67)
Supplement: Additional file 1 — Table S1. Description of myoblast and myotube samples for microarray analysis [file 1755-8794-4-67-S1.PDF]

Supplementary Table 1. Description of myoblast and myotube samples for microarray analysis

Name	Cell type	Age, gender of donor	Passage number	Source	Percent myoblasts[a]	Nuclei in myotubes[a] (%)	Size of pathogenic D4Z4 array (kb)	GEO ID
FM01	FSHD myoblasts	45 Y, F	P9	Biopsy (quadriceps)	98	-	27	GSE26145[b]
FM4	FSHD myoblasts	22 Y, F	P9	Scapula fixation (rhomboid)	95	-	23	GSE26145
FM9	FSHD myoblasts	13 Y, M	P9	Biopsy (deltoid)	90	-	18	GSE26145
FMD01	FSHD myotubes	45 Y, F	P9	Biopsy (quadriceps)	-	78	27	GSE26145
FMD4	FSHD myotubes	22 Y, F	P9	Scapula fixation (rhomboid)	-	75	23	GSE26145
FMD8	FSHD myotubes	14 Y, F	P9	Biopsy (quadriceps)	-	72	12	GSE26145
CM3	Normal-control myoblasts	27 Y, F	P9	Biopsy (quadriceps)	98	-	-	GSM443918
CM11	Normal-control myoblasts	23 Y, M	P9	Biopsy (quadriceps)	95	-	-	GSM443916
CM33	Disease-control myoblasts[c]	74 Y, F	P10	Biopsy (quadriceps)	94	-	-	GSM443914
CMD3	Normal-control myotubes	27 Y, F	P9	Biopsy (quadriceps)	-	80	-	GSM443917
CMD11	Normal-control myotubes	23 Y, M	P9	Biopsy (quadriceps)	-	76	-	GSM443915
CMD33	Disease control myotubes	74 Y, F	P10	Biopsy (quadriceps)	-	72	-	GSM443913

[a]The percentage of myoblasts was determined by desmin immunostaining and the percentage of nuclei in myotubes by combined desmin immunostaining and myosin heavy chain (MF20) immunostaining on an aliquot of each batch of cells used for microarray analysis. All the myotube preparations examined by expression profiling were from the same batch of myoblasts used for expression profiling except for FMD8. Although late-passage cells were examined so as to generate enough cells for both expression profiling and genome-wide DNaseI-hypersensitivity mapping (unpub. data), the cell batches used for profiling were capable of growing for an additional passage (1:3 or 1:4 splits) with >85% of the cells desmin-positive.

[b]In process for the GEO database.

[c]From moderately affected muscle of a patient with sporadic inclusion body myositis.
