# Supplementary material for: Gene expression during normal and FSHD myogenesis
Source: BMC Med Genomics. 2011 Sep 27;4:67. doi: 10.1186/1755-8794-4-67 (PMC3204225; doi:10.1186/1755-8794-4-67)
Supplement: Additional file 2 — Table S2. Descriptions of non-muscle cell types for expression profiling [file 1755-8794-4-67-S2.PDF]

Table S2. Descriptions of non-muscle cell types for expression profiling

Cell name and type (all human)	Gender of donor and age, if known	Number of biological replicates[a]	Source	GEO ID
Astrocytes (brain)	F	2	Lonza, CC-2565	GSM396630 / GSM396631
D721 (medulloblastoma, brain)	F, 2 Y	2	Surgical specimen, Duke University	GSM396632 / GSM396633
H54 (glioblastoma, brain)	M, 36 Y	4	Surgical specimen, Duke University	GSM396647 / GSM396648
GM12878 (lymphoblastoid cell)	F	3	Coriell, GM12878	GSM320159/GSM320160/GSM320161
K562 (myelogenous leukemia cells)	F, 53 Y	3	ATCC, CCL-243	GSM320148/GSM320149/GSM32150
Hepatocytes	F	1	ZenBio	GSM443933
HepG2 (hepatocellular carcinoma cells)	M, 15 Y	3	ATCC, HB-8065	GSM396652 / GSM396653 / GSM396654
LHSR (prostate epithelial cells)	M	2	Dana Farber Cancer Institute	GSM443935/ GSM443937
LnCAP (prostate cancer cells)	M, 50 Y	2	ATCC, CRL-1740	GSM443919 / GSM443920
HMEC (human mammary epithelial cells)	F	3	Lonza, CC-3150	GSM396655 / GSM396656
MCF7 (breast cancer cells)	F, 69	1	ATCC, HTB-22	GSM443923 / GSM443924
NHEK (human epidermal keratinocytes)	F	2	Lonza, CC-2501	GSM396659 / GSM396660
Melanocytes	M	4	Sciencell, 2200	GSM443944 / GMS443945 / GSM443946 / GSM443947
Chordoma (notochord remnant cancer cells	M, 27 Y	2	Chordoma Foundation	GSM443910 / GSM443911
Osteoblasts	F	2	Lonza, CC-2538	GSM396661 / GSM396662
FB8470 (fibroblasts)	F, 10 Y	2	Coriell, AG08470	GSM443908 / GSM443909
H1 ES (embryonic stem cells)	M	4	Cellular Dynamics, ENCODE tier 2 cell line	not publically available
Chorion (fetal membrane cells)	M	1	Surgical specimen, Duke University	GSM443912
HUVEC (umbilical vein endothelial cells)	M	3	Lonza, CC-2517	GSM396657 / GSM396658

[a]Individual batches of cells from the same human cell strain or cell line were used as biological replicates
