# Supplementary material for: Gene expression during normal and FSHD myogenesis
Source: BMC Med Genomics. 2011 Sep 27;4:67. doi: 10.1186/1755-8794-4-67 (PMC3204225; doi:10.1186/1755-8794-4-67)
Supplement: Additional file 3 — Table S3. Description of myoblast and myotube samples for qRT-PCR [file 1755-8794-4-67-S3.PDF]

Table S3. Description of myoblast and myotube samples for qRT-PCR

Name	Cell type	Age, gender of donor	Passage number[a]	Source	Size of pathogenic D4Z4 array (kb)
CM1	Control myoblasts	42 Y, M	P9	Biopsy (quadriceps)	-
CM3[b]	Control myoblasts	27 Y, F	P9	Biopsy (quadriceps)	-
CM4	Control myoblasts	27 Y, M	P8	Biopsy (quadriceps)	-
CM5	Control myoblasts	31 Y, M	P9	Unknown surgical sample	-
CM6	Control myoblasts	45 Y, M	P9	Biopsy (quadriceps)	-
CM7	Control myoblasts	46 Y, M	P9	Biopsy (quadriceps)	-
CM8	Control myoblasts	35 Y, M	P9	Biopsy (quadriceps)	-
CM9	Control myoblasts	34 Y, M	P9	Biopsy (quadriceps)	-
CM10	Control myoblasts	31 Y, F	P7	Biopsy (quadriceps)	-
CMD1	Control myotubes	42 Y, M	P9	Biopsy (quadriceps)	-
CMD3[b]	Control myotubes	27 Y, F	P9	Biopsy (quadriceps)	-
CMD4	Control myotubes	27 Y, M	P8	Biopsy (quadriceps)	-
CMD5	Control myotubes	31 Y, M	P9	Unknown surgical sample	-
CMD6	Control myotubes	45 Y, M	P9	Biopsy (quadriceps)	-
CMD7	Control myotubes	46 Y, M	P9	Biopsy (quadriceps)	-
CMD8	Control myotubes	35 Y, M	P9	Biopsy (quadriceps)	-
CMD9	Control myotubes	34 Y, M	P9	Biopsy (quadriceps)	-
CMD10	Control myotubes	31 Y, F	P7	Biopsy (quadriceps)	-
FM1	FSHD myoblasts	41 Y, M	P9	Biopsy (quadriceps)	28
FM4[b]	FSHD myoblasts	22 Y, F	P9	Scapula fixation (rhomboid)	23
FM5	FSHD myotubes	29 Y, M	P9	Gastronemius	17
FM6	FSHD myoblasts	52 Y, M	P8	Biopsy (quadriceps)	23
FM7	FSHD myoblasts	18 Y, F	P9	Biopsy (deltoid)	16
FM8	FSHD myoblasts	14 Y, F	P9	Biopsy (quadriceps)	12
FM10	FSHD myoblasts	41 Y, M	P7	Biopsy (quadriceps)	29
FM11	FSHD myoblasts	48 Y, M	P7	Biopsy (quadriceps)	17
FMD1	FSHD myotubes	41 Y, M	P9	Biopsy (quadriceps)	28
FMD4[b]	FSHD myotubes	22 Y, F	P9	Scapula fixation (rhomboid)	23
FMD5	FSHD myotubes	29 Y, M	P9	Gastronemius	17
FMD6	FSHD myotubes	52 Y, M	P9	Biopsy (quadriceps)	23
FMD7	FSHD myotubes	18 Y, F	P9	Biopsy (deltoid)	16
FMD9	FSHD myotubes	13 Y, M	P9	Biopsy (deltoid)	18
FMD10	FSHD myotubes	41 Y, M	P7	Biopsy (quadriceps)	29
FMD11	FSHD myotubes	48 Y, M	P7	Biopsy (quadriceps)	17

[a]Cell cultures were split 1:3 or 1:4 and grown as described in Materials and Methods. Desmin staining were done for all aliquots of the batches of myoblast samples used for qRT-PCR. Desmin and myosin heavy chain staining was done for an aliquot of the batch of cells used for qRT-PCR (FMD4, 5, 6, 7, 10, 11 and CMD1, 3, 5, 10) and or an aliquot from one passage earlier or later (FMD1, 9 and CMD 4, 6, 7, 8, 9). All myoblast samples were ≥85% desmin-positive, and all myotube preparations had ≥70% of the nuclei in desmin-positive, MF20-positive multinucleated myotubes (>2 nuclei per cell). In Fig. 1, of the main article were the following samples: top panel from left: FM4, FMD4, and FMD7; bottom panel from left: CM5, CMD3, and CMD5.

[b]Only these samples were also used for the microarray.
