# Supplementary material for: Gene expression during normal and FSHD myogenesis
Source: BMC Med Genomics. 2011 Sep 27;4:67. doi: 10.1186/1755-8794-4-67 (PMC3204225; doi:10.1186/1755-8794-4-67)
Supplement: Additional file 4 — Additional Methods: Generation, propagation, and differentiation of myoblast cultures from human skeletal muscle. [file 1755-8794-4-67-S4.PDF]

Additional Methods: Generation, propagation, and differentiation of myoblast cultures from human skeletal muscle

A) Generating myoblast cell strains by collagenase/dispase dissociation of muscle biopsy tissue

(Pavlath GK, and Gussoni E, Methods in Molecular Medicine vol107 Human Cell Culture Protocols, 2nd Ed. Edited by Picot J, Humana Press Inc.)

1. Aspirate the transport medium and wash the specimen with 10 ml of PBS.
2. Aspirate the PBS and replace with 5 ml more of PBS.
3. Pour the specimen into a sterile Petri dish and cut into small pieces using two sterile #10 scalpels.
4. Pipette up as much of the specimen as possible and transfer to a sterile 15-ml tube. Wash the plate with an additional 5 ml of PBS.
5. Let the specimen settle to the bottom of the tube. Aspirate off the PBS, sparing as much of the specimen as possible.
6. Pipette 10 ml of PBS onto the specimen. Cap the tube and shake well. Let the specimen fall to the bottom of the tube, then aspirate off the PBS
7. Repeat step 5 twice (for a total of three times).
8. Aspirate the PBS, add 1.6 ml of Dispase II solution, 2 ml of collagenase 4 and 50 µl of 0.2 M $CaCl_2$.
9. Incubate in clean 37°C water for 15 min.
10. Triturate (pipette up and down) 10 times, try to avoid producing foam, and return the tube to the clean 37°C water bath for an additional 15 min.
11. Repeat the trituration.
12. If the tissue does not appear disassociated (by eye), repeat the incubation and trituration.
13. Place a 100-µm nylon cell strainer on the top of a 50-ml tube and pre-wet the filter with PBS.
14. Pass the sample through the cell strainer and rinse the strainer with an additional 5 ml PBS.
15. Pellet the cells for 5 min at about 500 x g at room temperature, aspirate the supernatant and resuspend in 10 ml of Primary Culture Media (see below).
16. Pre-plate for 90 min in a T75 flask. This will allow contaminating fibroblasts to adhere, but the myoblasts will not.
17. Transfer the suspension to a new T75 flask; discard old flask.
18. On the 3rd or 4th day after initiating the culture, supplement the medium with 4 µl of bFGF in 1 ml of F10 medium; myoblasts may not have adhered at this stage and will not yet have a normal appearance.
19. For the first day or so, cells will appear rounded and may not be all adhered. It can take up to a week for cells to adhere and flatten. If original biopsy sample was small, you may not easily see any cells for a few days.
20. Supplement with fresh bFGF every 3-4 days. Change the medium for the 1st time after one week if small foci have developed. If not, leave up to 10 days before changing the medium, but continue to supplement with 4 µ ofl bFGF every 3-4 days. If it is necessary to perform the first medium change earlier than 1 week, pellet the spent medium to recover any non- adherent cells and resuspend the cells in fresh medium.
21. Subsequently, change the medium approximately 2 x per week until growth foci are seen. Do not allow these foci to overgrow.
22. Redistribute cells as foci develop. Aspirate medium and rinse with 10 ml of PBS, rock flask gently to cover and aspirate. Add 1 ml 0.1% trypsin, rock the flask to cover surface and either place in incubator or leave at room temperature. Cells will begin to detach within a couple of minutes. Gently tap side of flask and check under microscope that cells are detached. Add 10 ml of mediumand return to the incubator in same flask.
23. Grow until the culture is approx. 60-80% confluent. Trypsinize as before, split to 3-4 flasks (10 ml medium per flask). This is passage 1.
24. Grow cultures to 60-80% confluence. Trypsinize using 1ml of 0.1% trypsin per flask.When cells are detached inactivate trypsin with 10 ml of F10 media (no conditioned media) per flask. Pool media containing cells and pellet for 5 min at about 500 x g at room temperature to pellet the cells.

25. Aspirate the supernatant and carefully resuspend the cell pellet in freezing medium. Use 2-3 ml per flask depending on the degree of confluency, (aim for 8-10 vials per cell strain).This is passage 2. Aliquot into cryogenic vials putting 1ml/vial and freeze slowly (Nalgene Cryo freezing container, 5100-0001) at least overnight and up to a few days. Transfer to liquid nitrogen for long-term storage.

Materials for generation of myoblast cell strains

Transport medium

Muscle tissue should be placed in this at biopsy for transport to laboratory. If necessary tissue may be sent to laboratory by FedEx priority overnight with cold packs (blue ice not dry ice).

F-10 Nutrient Media Gibco 11550-043

10% FBS Gibco 26140-079

2% Pen/Strep Gibco 1514-122

Collagenase 4

Worthington Chemical (800-445-9603), CLS-4, 100mg lyophilized, dissolved in 25 ml sterile PBS. Store at -20°C.

Dispase II

Dispase II / Roche 04 942 078 001 5 x 1g, 0.99 u/mg. Use at 2.4 units/ml.

Primary culture medium (to establish the cell strain)

50% Maintenance media without bFGF (see below)

50% MRC-5 conditioned F10 medium (see below).

Then just before use, add,

basic fibroblast growth factor (bFGF) to a final concentration of 10 ng/ml (4 µl of working solution [1 µg/ml] per 10 ml)

and dexamethasone to a final concentration of 1 µM (10 µl of working solution [100 µM] per 10 ml)

MRC-5 conditioned F10 medium is prepared by overlaying a 70% confluent MRC-5 culture (normal fetal lung fibroblasts; ATCC, CCL-171) with 10 ml Maintenance media without bFGF or dexamethasone) and incubating overnight. The media are pooled, filtered through a 0.45-um filter and stored in 5-ml batches at -20^{0}C until use.

Maintenance medium

F-10 Nutrient Medium GIBCO 11550-043

20% FBS GIBCO 26140-079

1% Pen/Strep GIBCO 1514-122

10 ng/ml bFGF Promega G5071 or Atlanta Biologicals 15140-122.

Mix F10, FBS and Pen/Strep, filter through a 0.22-µm filter (Corning 430767). Store 4^0C.

Add bFGF at 4 µl per 10 ml as above.

Add dexamethasone 10 ul per 10 ml as above.

Trypsin

Use at 0.1% in PBS.

Stock is 0.25%, Gibco 25200-056.

-wash with PBS

-add 1 ml of 0.1% trypsin, incubate at 37^0C 1-5 min (usually closer to 1 min)

-gently tap to remove the cell sheet

-add 10 ml of medium to flask

-split this 10 ml between the required number of flasks, (should be 10 ml medium total in each flask)

Working solutions of bFGF and dexamethasone

See Part B, below.

Freezing medium

90% FBS

10% DMSO

Plastics

T75 flasks, Corning 430725

Cell strainer 100 um nylon, BD Falcon 352360.

B) Myoblast and myotube culture: basic techniques

Preparation

1. Store medium, trypsin and PBS at 4°C in small aliquots.
2. Warm the medium to 37°C before using and make sure that trays in the incubator are even to get as even as spread of myoblasts on plates as possible. Regularly check that the concentration of CO_2 in the incubator is 5% with a Fyrite gas analyzer.

3. Coat dishes with gelatin for all myoblast propagation as follows.

   a) Newly autoclaved 0.7% gelatin (AMR-9764-100G, Amresco, Solon, OH, USA) solution is dissolved by heating to 37°C in clean water for a few seconds before adding to the plates. To reuse the gelatin stock up to several times, melt at 37°C and sterile filter (0.22 µ).
   b) For a 100-mm dish, 150 µl of gelatin solution is added (for a T75 flask, 200 µl). Immediately, spread the gelatin very evenly on the flask bottom with a sterile cell scraper. Even spreading is critical to encourage even myoblast distribution on the plate.
   c) Leave the flask at room temperature for at least an hour for the gelatin solution to solidify.
   d) Before using the coated flask, warm it to 37°C.
   e) Use the same day or store at 4°C for up to a month in a tightly sealed bag.

4. Preparing and using stocks of basic fibroblast growth factor (bFGF) and dexamethasone.

   a) The bFGF stock is 100 µg/ml of 10 mM Tris-HCl, pH7.6, and is stored at -20°C and the dexamethasone phosphate stock is 4.4 mg dexamethasone sodium phosphate per ml (8.5 mM) and stored at RT.
   b) The working solution is 1 µg/ml bFGF & 100 µM dexamethasone in medium.
   c) Do not store the working solution for more than one week at 4°C.
   d) The final concentrations in the myoblast medium are 10 ng/ml bFGF & 1 µM dexamethasone.
   e) Add 1/100 volume of the working solution on the day that you add the medium to the cells.

General Procedure for Splitting Cell Cultures

1. Use 10 ml of F10 medium containing 20% fetal bovine serum, 100 U/ml penicillin, 100 µg/ml streptomycin, 10 ng/ml bFGF, and 1 µM dexamethasone for culturing myoblasts per 100-mm dish or T75 flask.
2. Check the cultures each and every day. When they become about 60-70% confluent, split them 1:3 or 1:4 (never more thinly). Do not let cultures become more than 80% confluent before splitting. Add the bFGF/dexamethasone to the culture medium immediately after splitting.
3. If the density of the culture is not even, it is critical to split it sooner than usual (1:2 to 1:3 depending on the overall confluency) so that myoblasts in the confluent patches do not go into an irreversible G0.
4. It should take about 4-5 days for the culture to grow back to ~70% confluence after a 1:4 split. If the cell growth slows down a little (*e.g.,* if the culture is only 40-50% confluent on day 4 after splitting), try to stimulate growth by adding bFGF/dexamethasone every day as above. Do not use the cultures if they fail to grow back to 70% confluence within an additional 3 days.

Details about trypsinization

1. When the myoblast cultures are 60-70% confluent (for a 100-mm dish, about 2 - 2.5 million cells), remove the medium from the flask. Use 10 ml of PBS to wash the cells.
2. Remove the PBS.
3. Add 2 ml of 0.1% trypsin to the dish or flask and distribute the trypsin evenly by tilting the dish or flask.
4. Incubate the cells at 37°C for 2 to 3 min (if needed, up to 5 min). Check the cells under the microscope for >95% detachment once per minute.
5. Tap the dish or flask, let the cells detach from the dish or flask.
6. Immediately after you see >95% detachment of cells, add 8 ml of fresh medium to the flask and mix well by shaking gently (or tapping the flask).
7. Disperse the cell suspension well by pipeting up and down to disrupt clumps but avoid creating bubbles. If you inadvertently leave some cell clusters, part of the monolayer will reach confluence in regions of the dish before the overall confluence is 60-70%. If dispersion with a serological pipette is insufficient, use a dedicated micropipettor with 1000-µl filter tip.
8. Aliquot the cell suspension into 3 or 4 labeled dishes or flasks coated with 0.7% gelatin and containing enough medium for a final volume of 10 ml
9. Add bFGF and dexamethasone to final concentrations of 10 ng/ml bFGF and 1 µM dexamethasone.
10. Incubate at 37°C with 5% CO_2.

Freezing myoblasts for viability:

1. Harvest cells at ~70% confluence, collect them in 50-ml plastic tube (2 - 3 vials can be made per T75 flask), and count the cell number using a hematocytometer.
2. Centrifuge the cells at 1000 rpm for 5 min.
3. Remove the supernatant, wash with PBS, and centrifuge at 1000 rpm for 5 min.
4. Remove the supernatant and resuspend the cells in freezing medium (10% DMSO/90% FBS) so that the final concentration is 10^6 cells/ml of freezing medium.
5. Transfer 1 ml of the cell suspension in into each cryovial and keep them in a freezing container at -80°C overnight.
6. Next day, move cryovials to a liquid nitrogen tank.

Thawing myoblasts

Prepare a Styrofoam or plastic bucket with clean 37°C water in a chemical hood.

Thaw quickly in water and immediately put in a T75 that already was coated with gelatin and has 20 ml of medium.

Replace medium on the next morning to remove the DMSO.

Differentiation to myotubes

1. When the cell cultures are >95% confluent, remove the F-10 medium/bFGF/dexamethasone and wash with 10 ml of PBS (2 times).
2. Add 10 ml of DMEM containing 2% horse serum (HS), 2 mM L-glutamine, 100 U/ml penicillin, and 100 µg/ml streptomycin.
3. After 24 h of incubation, increase the HS concentration by replacing the 2% HS medium with 10 ml of DMEM containing 15% HS, 2 mM L-glutamine, 100 U/ml penicillin and 100 µg/ml streptomycin.
4. Leave the dishes in the incubator until using cells for harvesting at the appropriate time points. For maximal myotube formation, this is usually 5 - 7 days for biopsy-derived myoblasts and 4 - 5 days for fetal myoblasts.

Materials for myoblast culture

F10 Medium: Invitrogen Cat#11550043

Penicillin/streptmycin: Invitrogen Cat#15140122

Fetal bovine serum (FBS): Hyclone

Basic fibroblast growth factor (bFGF, human recombinant): 10 µg powder from Invitrogen Cat#13256029; dissolve powder in 100 µl of 10 mM Tris-HCl, pH 7.6 to 100 ng/µl and store at -20°C.

Dexamethasone sodium phosphate, (516 MW, 4 mg/ml as dexamethasone phosphate) (American Regent Inc. NDC 0517-4901-25) . Store at room temperature.

Trypsin: dilute to 0.1% trypsin from 0.25% trypsin (Trypsin, 0.25% (1X) with EDTA 4Na, liquid: Invitrogen Cat#25200056) with 1x PBS

Dulbecco's Modified Eagle Medium (DMEM): Invitrogen Cat#11960069

Horse serum: Invitrogen Cat#16050-130

L-glutamine (200 mM 10x): Invitrogen Cat#25030081

Desmin immuno-staining and nuclei staining

Note that myoblast cultures routinely contain fibroblast-like cells and that as the myoblast cell strain is passed (especially in late passages, more than P8 with 1:4 splits) or under less than optimal growth conditions, the percentage of non-myoblast cells sometimes increases to >15% fibroblast-like cells, which can only be detected by immunostaining. In addition, there may be an accumulation of myotubes, especially if the cells are not under optimal growth conditions or if some areas in the flask or dish have cells at >80% confluence. Therefore, for every experiment, the quality of the myoblast preparation of that batch should be checked by desmin staining on cells growing in the same type and size of

dish used for the experiment. It is difficult to distinguish fibroblasts from myoblasts without immunostaining. The culture should contain ~80-95% of nuclei in desmin-positive cells.

For checking differentiation of cells to myotubes, the desmin immunostaining can be used to determine the average number of nuclei per cell. The multinucleated status of myotubes can be used to identify nuclei in myotubes. The percent differentiation can be expressed as nuclei in cells with more than 2 nuclei/total nuclei. In addition, the desmin staining of the myotubes is even stronger than that of the myoblasts. The myoblast preparation should contain at least 70% of nuclei in myotubes (cells with more than 2 nuclei) at the day of harvesting (day 5-8 after first adding the differentiation medium).

To check the percentage of myoblast by desmin immunostaining, prepare a 35-, 50-, or 100-mm dish for staining concurrently with the cells plated for the main experiment.

The whole procedure can be done at room temperature.

1. Use PBS to wash the cells once. Then aspirate the PBS solution.
2. Make a circle the same size as your cover glass (22 mm) using a Pap pen (wax crayon pen). All procedures from step3 through step12 will be done within the circled area).
3. Add 2% paraformaldehyde solution to fix the cells for 15 min. The volume of the paraformaldehyde solution should be enough to cover the circled section of the dish that you want to stain. Keep the paraformaldehyde solution on ice; it is important that it be cold when added.
4. Aspirate the paraformaldehyde solution. Add 0.25% Triton solution to permeabilize the cell membrane and nuclei membrane for 20 min.
5. Treat the cells with blocking solution PBS+BSA+azide (PBS, 1 mg/ml BSA, 10 mM Na azide) for 30 min. The blocking solution should cover the whole circled section of the dish that you want to stain.
6. Add the working solution of 1° antibody, rabbit anti-desmin to the circled area on the dish. For the primary antibody working solution, dilute the 1° antibody to 1:100 with PBS+BSA+azide solution to make a 100-µl working solution. (For double staining with desmin and MF20, add 100 µl of the working solution mixture of desmin and MF20 which are diluted to 1:100 with PBS+BSA+Azide solution.) This solution should cover the circled region. Let the cells incubate with 1° antibody for 1 h at room temperature.
7. Wash the cells with PBS+BSA+azide solution 3 times, leaving the solution on the cells 15 min for each wash.
8. Add the working solution of the 2° antibody, anti-rabbit IgG antibody. The solution should cover the circled region. Incubate at room temperature for 1 h. (For double staining with desmin and MF20, add the working solution of the 2° antibody, anti-rabbit IgG antibody for desmin anti-mouse IgG2b for MF20.)
9. Repeat the wash process as described in step 7.
10. Aspirate the wash solution. Add 100 µl of 50 µg/ml propidium iodide (or for staining with MF20, use a 1:1000 dilution of DAPI instead) to the cells and incubate at room temperature for 10 min.
11. Repeat washing as described in step 6, but with 5 min instead of 15 min.
12. Mount the cover slip to a microscope slide by using Vectashield H-1000 mounting medium for fluorescence (Vector laboratories, Inc. Burlingame, CA) and tape the slip with nail protector (Available in supermarket). Check the cells under the fluorescence microscope.
13. Store slides at 4°C.

Reagents and solutions for desmin immunostaining and counterstaining of nuclei

Primary antibody: rabbit anti-desmin, 200 µg/µl, RB-9014-p0, Labvision, CA; make a working solution for addition to cells by diluting the stock 1:100 in 200 µl the PBS+BSA+azide solution. Make sure that any new batch of desmin antibody is specific for myoblasts by immunostaining a fibroblast culture, which should be negative.

Secondary antibody: anti-rabbit IgG antibody Glexa 488 goat anti-rabbit A-11034, Molecular probe, CA; make a working solution for addition to cells by diluting the stock 1:100 in 200 µl the PBS+BSA+azide solution.

2% paraformaldehyde solution: Weight out 2 g of paraformaldehyde (Sigma Cat#P6148) powder. Transfer the paraformaldehyde to a beaker with 80 ml water. Heat and stir the solution in a chemical hood until the paraformaldehyde dissolve. Make the final volume 100 ml by adding more water. Filter the paraformaldehyde solution before it turns cool. Store the solution at 4°C.

0.25% Triton solution: Dissolve 250 µl of Triton X-100 (Sigma) to 100 ml water. Store at room temperature.

PBS+BSA+azide (1X PBS, 1 mg/ml BSA, 10 mM Na azide) solution: dissolve 8.5 g NaCl (Sigma), 1.38 g NaH2PO4 (Sigma), 0.65 g NaN3 (Sigma) and 1 g of BSA (Sigma) in 1 L of water. Store at 4°C.

50 µg/ml propidium iodide solution: 5 mg of propidium iodide (Sigma) in 100 ml of water. Store in the dark at 4°C.

DAPI (4',6'-diamidino-2-phenylindole•2HCl, Invitrogen): 1 mg/ml stock in water. Store in the dark at 4°C. The 1:1000 dilution is made just before use with water.
