# Supplementary material for: Gene expression during normal and FSHD myogenesis
Source: BMC Med Genomics. 2011 Sep 27;4:67. doi: 10.1186/1755-8794-4-67 (PMC3204225; doi:10.1186/1755-8794-4-67)
Supplement: Additional file 5 — Table S4. qPCR primers and full names of genes tested. [file 1755-8794-4-67-S5.PDF]

Table S4. qPCR primers and full names of genes tested

Gene		Forward primer	Reverse primer	Annealing temp.	Amplicon size (bp)	RefSeq	Location
PCOLCE	Procollagen C-endopeptidase	GACTGCGACAGTGAAGTCCA	GCTTGCAAGGCACGTAAAAC	58	146	NM_002593	Ex8
RUVBL2	RuvB-like 2 (E. coli)	CCAAGATGATTGAGTCCCTGAC	GCACGAACTTGGTCTGG[a]	60	150	NM_006666	Ex7-9
TGFB2	Transforming growth factor, beta 2	AATTGCTGCCTACGTCCACT	TTGGGTTCGTGTATCCATTTC	62	70	NM_00113559[illegible]	Ex6
EIF2C2	Eukaryotic translation initiation factor 2C, 2	TGAGATGACAGACGTGACTGG	CAGGGGTCGCAATAGCTTTA	60	83	NM_012154	Ex10-11
BGN	Biglycan	CTCCCCTGACACCACGCTGCT	AGGTGCTGGAGACCCTTGAAGTCAT	58	84	NM_001711	Ex3
DCN	Decorin	CCTTCACGCATTGATTCTTGT	AAAGTCGTTCCAACTTCACCA	58	86	NM_133503	Ex3-4
MYOM1	Myomesin 1, 185kDa	CTCTGAGGGCTCACCAACTC	AAAACTGGCAGCGAGTGAAC	62	134	NM_003803	Ex15
SFRS9	Serine/arginine-rich splicing factor 9	GATGCGGCCGTACTTGTAGA	GCATCTACGTGGGGAACCT	60	77	NM_003769	Ex1
CAPG	Capping protein (actin filament), gelsolin-like	CCCAGAAGAGGTTTCCCATC	CTCTCCCAGCAGCGTGTT	60	109	NM_001747	Ex3-4
IGFBP6	Insulin-like growth factor binding protein [illegible]	GCCGTAGACATCTGGACTCAGTGCT	TGGGCACGTAGAGTGTTTGAGCC	62	78	NM_002178	Ex3
FRAS1	Fraser syndrome 1	GAGCAGTTCCTCAACCTCGT	TCCCAGTGTTGTGGAGATCA	60	85	NM_025074	Ex20-21
M6PR	Mannose-6-phosphate receptor (cation dependent)	GGGAGCCAAAGGAATGGAGCAGT	TGCTGCAGGCACATTTCGAGGT	60	118	NM_002355	Ex6-7

[a]This was the only reaction using FAM reporter technology. The probe for *RUVBL2* was TACGACGCTATGGGCTCC (Solaris). The other reactions used SYBR-Green reporter.
