# Supplementary material for: Gene expression during normal and FSHD myogenesis
Source: BMC Med Genomics. 2011 Sep 27;4:67. doi: 10.1186/1755-8794-4-67 (PMC3204225; doi:10.1186/1755-8794-4-67)
Supplement: Additional file 6 — Figure S1. FSHD-downregulated genes: relationship between gene expression in FSHD myotubes vs. non-muscle cell types to that in control myotubes vs. non-muscle cell types. [file 1755-8794-4-67-S6.PDF]

Figure S1

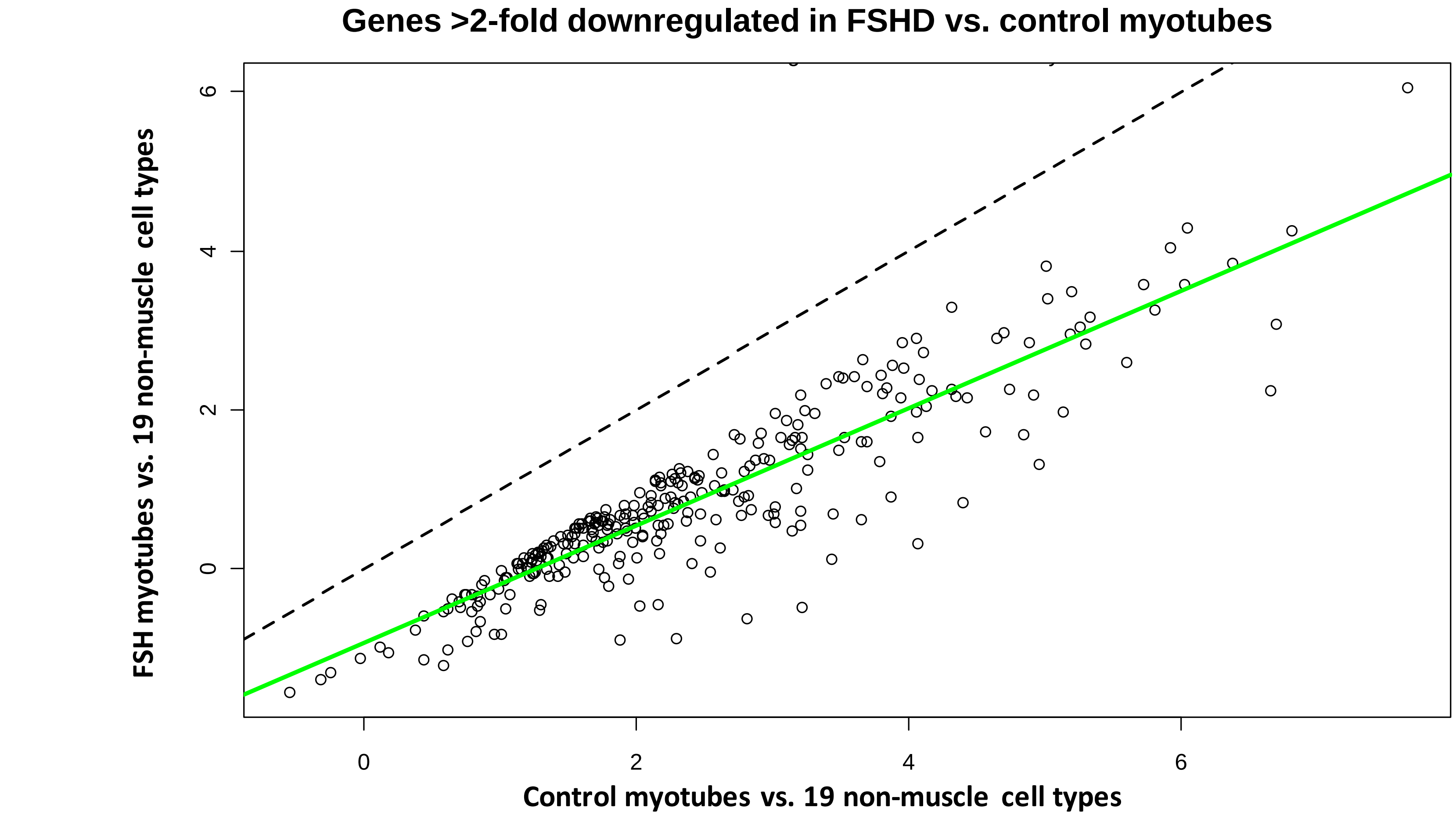

FSHD-downregulated genes: relationship between gene expression in FSHD myotubes vs. non-muscle cell types to that in control myotubes vs. non-muscle cell types. Only genes with >2-fold downregulation of expression in FSHD vs. control myotubes (*p*<0.01) are shown. The fitted regression equation is y = -0.94 + 0.74x, where x = Ctl Mt vs non-muscle cells and y = FSHD Mt vs non-muscle cells. The R^2 coefficient for the model is 0.82. The fitted line is shown in green, with the dashed y=x line included for reference. This figure illustrates that many of the genes with downregulation in FSHD vs. control myotubes were still more highly expressed in FSHD myotubes than in the 19 non-muscle cell types subject to identical expression profiling.
