# Supplementary material for: Gene expression during normal and FSHD myogenesis
Source: BMC Med Genomics. 2011 Sep 27;4:67. doi: 10.1186/1755-8794-4-67 (PMC3204225; doi:10.1186/1755-8794-4-67)
Supplement: Additional file 7 — Figure S2. FSHD-upregulated genes: relationship between gene expression in FSHD myotubes vs. non-muscle cell types to that in control myotubes vs. non-muscle cell types. [file 1755-8794-4-67-S7.PDF]

Figure S2

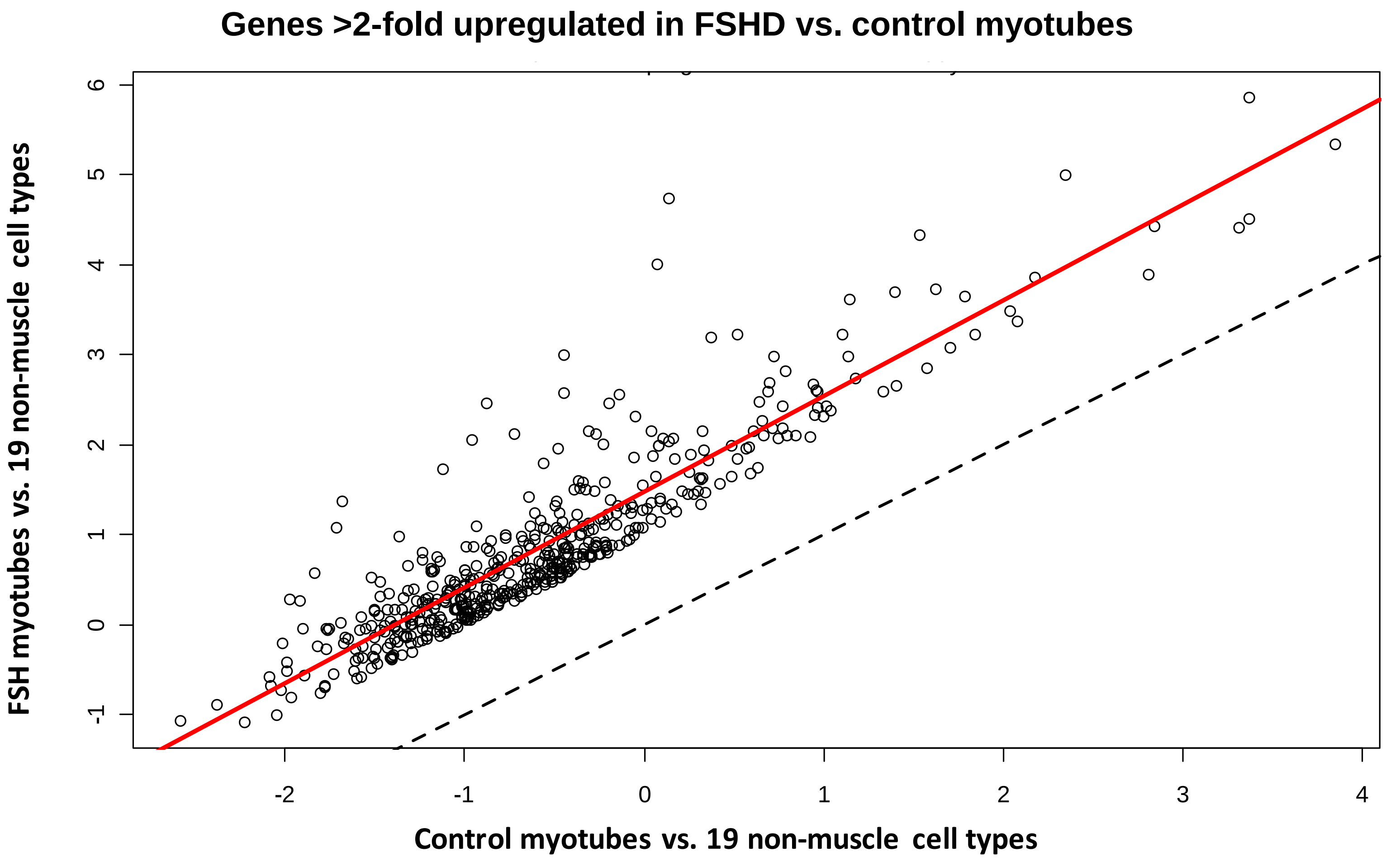

FSHD-upregulated genes: relationship between gene expression in FSHD myotubes vs. non-muscle cell types to that in control myotubes vs. non-muscle cell types. This figure is analogous to the last one except that it shows genes with >2-fold upregulation ($p<0.01$) in FSHD vs. control myotubes. Fitted regression equation: y= 1.47 + 1.06x, where x = CTL Mt vs non-muscle and y = FSHD Mt vs non-muscle. The R^2 coefficient for the model is 0.82. The fitted line is shown in red, with the dashed y=x line included for reference. This figure illustrates that only a small percentage of the genes with upregulation in FSHD vs. control myotubes were downregulated in FSHD myotubes vs.19 non-muscle cell types subject to identical expression profiling.
