# Supplementary material for: Gene expression during normal and FSHD myogenesis
Source: BMC Med Genomics. 2011 Sep 27;4:67. doi: 10.1186/1755-8794-4-67 (PMC3204225; doi:10.1186/1755-8794-4-67)
Supplement: Additional file 8 — Table S5. Some pathways and functional terms overrepresented among FSHD-dysregulated genes. [file 1755-8794-4-67-S8.PDF]

Table S5. Some pathways and functional terms overrepresented among FSHD-dysregulated genes

Functional terms[a]	Mb or Mt[b]	No. of up- or dnreg. genes	Ratio[c]	FSHD-dysregulated genes
RNA-induced silencing complex, GO:0035278 (5 genes)	Mt	4 dn	0.80	*EIF2C1, EIF2C2, EIF2C3, EIF2C4*
	Mb	1 dn	0.20	*EIF2C2*
Fatty acid elongation in mitochondria, IPA (16 genes)	Mb	7 up	0.44	*HADH, HADHA, HSD17B10, MECR, PECR, PPT1, PPT2*
	Mt	2 up	0.13	*HSD17B10, MECR*
Extracellular matrix, GO:0031012 (121 genes)	Mt	35 up & 1 dn	0.30	Up: *AEBP1, ASPN, BGN, COL15A1, COL1A1, COL1A2, COL21A1, COL3A1, COL4A2, COL5A1, COL5A2, COL6A1, COL6A2, COL6A3, DCN, DPT, EFEMP1, EMILIN1, FBLN1, FBLN5, FMOD, IGFBP7, LGALS1, LGALS3BP, LMCD1, LOXL1, LTBP1, LTBP2, MFAP4, MGP, NID2, PCOLCE, SOD1, TGFB1I1, VCAN;* Dn: *TGFB3*
	Mb	4 up & 2 dn	0.05	Up: *BGN, COL6A1, PCOLCE, TGFB1I1* ; Dn: *COL12A1, TGFB2*
NRF2-mediated oxidative stress response, IPA (165 genes)	Mt	15 up, 7 dn	0.13	Up: *ACTG1, CLPP, DNAJB1, DNAJC1, DNAJC4, GSTA4, GSTP1, JUNB, MAP2K2, PMF1, PPIB, PRKCD, RBX1, RRAS, SOD1* ; Dn: *DNAJA4, DNAJC3, GSK3B, MAPK8, PIK3C2B, PIK3R3, PRKCQ*
	Mb	7 up	0.04	*DNAJB6, DNAJC4, GSTP1, KEAP1, MGST3, PMF1, RBX1*
HIF1α Signaling, IPA (105 genes)	Mt	6 up, 7 dn	0.12	Up: *HSP90AA1, PGF, RBX1, RRAS, VEGFA, VEGFB* ; Dn: *MAPK8, NCOA1, PIK3C2B, PIK3R3, SLC2A4, SLC2A5, HIF1AN*
	Mb	5 up	0.05	*HSP90AA1, PGF, RBX1, TCEB2, VEGFB*
Regulation of actin-based motility by Rho, IPA (88 genes)	Mt	9 up, 3 dn	0.10	Up: *ARPC1B, MYL6, PPP1CA, RHOA, RHOC, RHOD, RHOG, RHOJ, RND2;* Dn: *MYL1, PI4KA, PPP1R12B*
	Mb	6 up	0.07	*ARPC1B, CFL1, MYL6, MYL6B, PPP1CA, RHOA*
Mitochondrial matrix GO: 0005759 (214 genes)	Mt	18 up & 5 dn	0.11	Up: *ALDH2, ATP5D, CLPP, DCI, DGUOK, GLUD1, GOT2, HMGCL, HSD17B10, HSD17B8, IDH1, IDH3B, IDH3G, MDH2, PCK2, SHMT2, SOD1, TST* ; Dn: *BCKDHB, CASQ1, ALDH5A1, PDK4, MAS1*
	Mb	22 up & 1 dn	0.11	Up: *ACADVL, ATP5D, BAT1, CARS2, COQ3, DGUOK, ETFB, ETFDH, GOT2, HADH, HADHA, HMGCL, HSD17B10, HSD17B8, IDH3B, IDH3G, MDH1, MDH2, PMPCB, PRDX1, SARDH, YARS2* ; Dn: *ACSM5*
Oxidative phosphorylation, IPA (116 genes)	Mt	18 up	0.16	Up: *ATP5D, ATP5I, ATP6V0B, ATP6V0E1, COX6B1, COX7A2, COX7A2L, NDUFA11, NDUFA12, NDUFA2, NDUFA4L2, NDUFB11, NDUFB2, NDUFB8, NDUFC1, NDUFS3, NDUFS8, SDHB*
	Mb	11 up	0.09	Up: *ATP5D, ATP5I, ATP5J, COX6B1, NDUFA2, NDUFA4L2, NDUFB2, NDUFB8, NDUFC1, NDUFS2, NDUFS3*
Induction of apoptosis, GO:0006917 (174 genes)	Mt	13 up, 5 dn	0.09	Up: *BAD, EI24, INHBA, NDUFS3, NME3, NUDT2, NUPR1, PLEKHF1, PML, PYCARD, TGFB1, TNFRSF25, TRADD* ; Dn: *DAPK1, FEM1B, PRUNE2, TGFB3, ZMAT3*
	Mb	8 up, 2 dn	0.05	Up: *CDKN2C, CIDEC, EI24, NDUFS3, NUDT2, TGFB1, TNFRSF10A, TRADD;* Dn: *DAPK1, TGFBR1*
Anti-apoptosis, GO:0006916 (188 genes)	Mt	16 up, 3 dn	0.10	Up: *ANGPT1, ANXA5, API5, CCL2, CLU, DAD1, GSTP1, HBXIP, HSPB1, IGF1, SOD1, SPHK1, TXNDC5, UBA52, VEGFA, VEGFB;* Dn: *HIPK3, NGFR, TRAF6*
	Mb	4 up	0.02	Up: *CFL1, GSTP1, HBXIP, VEGFB*

[a] Overrepresented functional terms for genes up- or downregulated in FSHD vs. control cells (p <0.01) were identified by bioinformatics programs, DAVID (http://david.abcc.ncifcrf.gov/ for GO terms) or IPA (http://www.ingenuity.com/). The number of genes on the microarray associated with the functional term is given in parentheses. The total number of up- or downregulated genes in FSHD vs. control myoblasts or myotubes (p <0.01, no fold-change threshold) were 524 and 48 (for myoblasts) and 914 and 466 (for myotubes), respectively. The data in this table supplement those of Table 3.

[b] Mb, Mt: myoblasts, myotubes

[c] The number of genes for a given pathway or GO-term that were up- or downregulated in FSHD vs. control myogenic cells/the total number of genes for that pathway or GO-term that were included in the microarray
