# Supplementary material for: Gene expression during normal and FSHD myogenesis
Source: BMC Med Genomics. 2011 Sep 27;4:67. doi: 10.1186/1755-8794-4-67 (PMC3204225; doi:10.1186/1755-8794-4-67)
Supplement: Additional file 9 — Table S6. Most of the pro- or anti-apoptotic genes that were dysregulated in FSHD vs. control myotubes were apparently up- or downregulated in FSHD myotubes because of dampening of their normal myogenesis-associated changes in expression. [file 1755-8794-4-67-S9.PDF]

Table S6. Most of the pro- or anti-apoptotic genes that were dysregulated in FSHD vs. control myotubes were apparently up- or downregulated in FSHD myotubes because of dampening of their normal myogenesis-associated changes in expression

		Fold change [a]	
Gene	Gene description	FSH myot / Ctl myot	Ctl myot/ Non-muscle
Inducer of apoptotsis, GO:000691			
NUPR1	nuclear protein, transcriptional regulator, 1	*3.4*	-1.5
INHBA	inhibin, beta A	*3.1*	1.9
EI24	etoposide induced 2.4 mRNA	2.2	-1.2
TRADD	TNFRSF1A-associated via death domain	*2.2*	*-2.0*
NDUFS3	NADH dehydrogenase (ubiquinone) Fe-S protein 3, 30kDa (NADH-coenzyme Q reductase)	2.1	-1.6
NUDT2	nudix (nucleoside diphosphate linked moiety X)-type motif 2	2.1	-1.6
TGFB1	transforming growth factor, beta 1	*2.0*	*-2.2*
PLEKHF1	pleckstrin homology domain containing, family F (with FYVE domain) member 1	1.9	-1.3
NME3	non-metastatic cells 3, protein expressed in	*1.8*	*-1.9*
PML	promyelocytic leukemia	*1.8*	*-1.5*
PYCARD	PYD and CARD domain containing	1.7	*-2.1*
BAD	BCL2-associated agonist of cell death	1.6	-1.9
TNFRSF25	tumor necrosis factor receptor superfamily, member 25	1.6	-1.1
PRUNE2	prune homolog 2 (Drosophila)	-3.3	*65.5*
DAPK1	death-associated protein kinase 1	*-2.6*	*4.3*
TGFB3	transforming growth factor, beta 3	*-2.2*	*5.0*
FEM1B	fem-1 homolog b (C. elegans)	*-1.7*	*1.5*
ZMAT3	zinc finger, matrin type 3	-1.6	*5.1*
Anti-apoptosis, GO:000691			
CCL2	chemokine (C-C motif) ligand 2	*7.0*	*1.53*
GSTP1	glutathione S-transferase pi 1	*2.9*	-0.84
CLU	clusterin	2.5	*-1.61*
VEGFA	vascular endothelial growth factor A	2.5	-0.56
UBA52	ubiquitin A-52 residue ribosomal protein fusion product 1	*2.5*	*-1.26*
ANGPT1	angiopoietin 1	2.4	*1.57*
DAD1	defender against cell death 1	2.1	-0.32
IGF1	insulin-like growth factor 1 (somatomedin C)	2.0	-0.19
VEGFB	vascular endothelial growth factor B	*2.0*	*-0.89*
HBXIP	hepatitis B virus x interacting protein	1.9	-0.60
SOD1	superoxide dismutase 1, soluble	1.9	-0.52
HSPB1	heat shock 27kDa protein 1	1.9	*-0.68*
SPHK1	sphingosine kinase 1	*1.8*	*-0.95*
ANXA5	annexin A5	1.7	*0.96*
API5	apoptosis inhibitor 5	1.7	-0.33
TXNDC5	thioredoxin domain containing 5 (endoplasmic reticulum)	1.7	*-0.60*
TRAF6	TNF receptor-associated factor 6	*-2.4*	*1.24*
HIPK3	homeodomain interacting protein kinase 3	-2.3	*3.09*
NGFR	nerve growth factor receptor	-2.1	*1.70*

[a] The genes associated with the above two GO terms that were significantly dysregulated in FSHD vs. control myotubes are shown. Similar results were obtained for genes in these two GO terms dysregulated in FSHD vs. control myoblasts. Fold changes in RNA signal for FSHD cells vs. non-muscle cells were determined by identical expression profiling of FSHD and control myogenic cells and 19 non-muscle cell types (See Additional File 1 and Additional File 2 for details about cell samples). Green highlighting, significant FSHD downregulation (p <0.01); pink, significant FSHD upregulation (p <0.01 or, if in italics, p <0.001).
